# Generational safety profiles of BTK inhibitors: adverse reaction signals and real-world evidence from the FAERS database

**DOI:** 10.3389/fmed.2026.1832040

**Published:** 2026-09-04

**Authors:** Jiachen Tu, Zhe Wang, Jingjing Zhang, Haojie Xu, Yanming Li, Li Zhang, Jian Gong, Libo Zhao

**Affiliations:** 1 Department of Pharmacy, Peking University Third Hospital, Beijing, China; 2 Department of Pharmacy, Wuhan Huangpi District People's Hospital, Wuhan, China; 3 Department of Pharmacy, Beijing Children's Hospital, Capital Medical University, Beijing, China; 4 Department of Pharmacy Administration and Clinical Pharmacy, Peking University, Beijing, China; 5 Research Group of Jian Gong on Pharmacoepidemiology and Clinical Drug Evaluation, School of Clinical Pharmacy, Shenyang Pharmaceutical University, Shenyang, China

**Keywords:** adverse events, BTK inhibitors, FAERS database, generational differences, real-world studies

## Abstract

**Background:**

The development of Bruton's tyrosine kinase (BTK) inhibitors has revolutionized the management of B-cell malignancies and holds potential in autoimmune diseases. However, safety concerns remain regarding treatment-associated adverse events: first-generation ibrutinib has been associated with off-target adverse event profiles, second-generation agents (acalabrutinib and zanubrutinib) require further validation of long-term safety patterns, and the real-world safety characteristics of third-generation pirtobrutinib continue to be evaluated. This study aims to characterize differences in adverse event (AE) disproportionality reporting signal patterns among four BTK inhibitors using data from the FDA Adverse Event Reporting System (FAERS).

**Methods:**

This study analyzed AE reports for four BTK inhibitors from the FAERS database via disproportionality analysis with four distinct algorithms, complemented by a clinical prioritization scoring system. Disproportionality reporting signal patterns were characterized across System Organ Class (SOC), Preferred Term (PT), and temporal distribution dimensions. A standardized 2-year observation window was applied for sensitivity analysis, and AE-mortality associations were evaluated.

**Results:**

A total of 77,014 AE reports were included in this study. The four BTK inhibitors showed positive disproportionality reporting signals in blood and lymphatic system disorders, cardiac disorders, and infections and infestations. However, distinct disproportionality signal patterns were observed at the PT level. Ibrutinib showed prominent cardiovascular-related disproportionality reporting signals, particularly for atrial fibrillation (ROR = 9.8). Acalabrutinib was characterized by a distinct headache signal. Zanubrutinib showed prominent signals for myelosuppression (ROR = 16.24) and haemorrhage subcutaneous (ROR = 146.81). Pirtobrutinib demonstrated the strongest signal for increased white blood cell count (ROR = 54.28). Descriptive analysis of reported time-to-onset distributions showed that pneumonia reports for ibrutinib represented a larger proportion among cases reported after longer treatment durations, whereas haemorrhage and decreased white blood cell count reports were more frequently observed within earlier reporting intervals. In terms of fatal outcomes, multiple PTs associated with ibrutinib showed statistically significant associations with reported death outcomes within the FAERS dataset.

**Conclusion:**

This descriptive pharmacovigilance study identified distinct disproportionality reporting signal patterns for four BTK inhibitors based on FAERS data, providing potential signals for post-marketing safety surveillance. These findings should be interpreted as differences in reporting patterns.

## 1 Introduction

B-cell receptor (BCR) signaling pathway (1), plays a central role in the development and progression of B-cell malignancies. As a non-receptor tyrosine kinase in the Tec family, BTK mediates key immunological processes including antigen presentation, cytokine production, and immune synapse formation, making it a pivotal regulator of humoral immunity (2, 3). BTK is expressed during B-cell differentiation, and its dysregulated activation promotes aberrant B-cell proliferation and survival (4), driving tumorigenesis in chronic lymphocytic leukemia (CLL) (5), aggressive progression in mantle cell lymphoma (MCL) (6), and abnormal monoclonal immunoglobulin secretion in Waldenström's macroglobulinemia (WM) (7). The introduction of the first-generation BTK inhibitor ibrutinib marked a therapeutic breakthrough in the management of B-cell hematologic malignancies, particularly CLL. This class of agents induces apoptosis in malignant B cells by selectively inhibiting BTK and suppressing BCR signaling (8). As a first-generation BTK inhibitor, ibrutinib has demonstrated superior progression-free survival (PFS) and overall survival (OS) compared to conventional chemotherapy (9). The subsequent development of second-generation BTK inhibitors acalabrutinib demonstrated comparable clinical efficacy to ibrutinib, with differences in adverse event profiles reported in clinical studies (10). Similarly, the ALPINE trial demonstrated that zanubrutinib achieved improved efficacy outcomes in clinical trials, accompanied by differences in reported adverse event patterns compared with ibrutinib (11), leading to guideline endorsement (12). Most recently, pirtobrutinib, a third-generation BTK inhibitor approved in 2023, exhibits non-covalent binding that circumvents resistance mediated by Cys481 mutations (13). It is indicated for patients with relapsed/refractory CLL/small lymphocytic lymphoma (SLL) who have received at least two prior lines of therapy including BTK inhibitors (14). Critically, BTK is also expressed in myeloid cells and contributes to pathogenic signaling in autoimmune conditions (15) such as rheumatoid arthritis (RA), systemic lupus erythematosus (SLE), and multiple sclerosis (MS) (16), positioning BTK inhibition as a promising therapeutic strategy for immune-mediated disorders. This study focuses on four BTK inhibitors: ibrutinib, acalabrutinib, zanubrutinib, and pirtobrutinib. They are the four FDA-approved BTK inhibitors for treating B-cell malignancies, are widely used in clinical practice, and thus their safety is of significant concern. They represent the generational evolution from first- to third-generation BTK inhibitors, providing an ideal framework for analyzing potential shifts in their safety profiles. Furthermore, all four agents have accumulated substantial post-marketing reports in FAERS, enabling descriptive post-marketing signal detection across different generations of BTK inhibitors. A systematic characterization of their disproportionality reporting signal patterns may help identify potential safety signals and generate hypotheses for further clinical evaluation.

Despite their established efficacy, the widespread clinical adoption of BTK inhibitors has been tempered by significant safety concerns. The first-generation inhibitor ibrutinib, primarily due to its off-target kinase activity (17), is associated with a higher reported frequency of AEs such as atrial fibrillation, haemorrhage and infections (18, 19). Although second-generation BTK inhibitors, including acalabrutinib and zanubrutinib, were designed for greater selectivity, their long-term safety data remain limited and necessitate further validation through real-world evidence. Furthermore, the emergence of resistance, notably via the BTK C481S mutation, represents a major therapeutic challenge (20). Pirtobrutinib, a third-generation non-covalent inhibitor, provides a promising option for patients refractory to prior BTK inhibitor therapy (21). However, the comprehensive safety spectrum associated with this newest agent are not yet fully elucidated. These treatment-related toxicities frequently result in therapy discontinuation, significantly impair patients' quality of life, and ultimately compromise long-term therapeutic success. Consequently, there is a pressing clinical need for a systematic post-marketing signal detection across the entire class of BTK inhibitors. The current evidence base predominantly consists of isolated studies on individual agents (22, 23) or head-to-head comparisons limited to two agents (24). This fragmented approach has hindered the development of a unified analytical framework capable of simultaneously evaluating all available BTK inhibitors.

The U.S. Food and Drug Administration (FDA) Adverse Event Reporting System (FAERS), a cornerstone of global post-marketing surveillance, represents one of the world's largest repositories of spontaneously reported adverse drug events. The database's principal strengths lie in its vast sample size, diverse population coverage, and well-established utility in identifying rare and unexpected adverse reaction signals (25). These attributes make FAERS a valuable resource for identifying potential post-marketing safety signals and exploring reporting patterns associated with BTK inhibitors. Nevertheless, existing pharmacovigilance studies utilizing FAERS data to investigate BTK inhibitors are subject to considerable methodological limitations. A predominant issue is their reliance on a single disproportionality analysis algorithm or a narrow focus on a selective set of AEs (26–31). Such a constrained analytical scope often overlooks the strategic application of multiple, complementary signal detection methodologies, which is essential for verifying the consistency and robustness of any identified safety signals. To overcome these constraints, this study conducted a comprehensive descriptive pharmacovigilance analysis of four BTK inhibitors based on the FAERS database. The application of four distinct signal detection algorithms enabled cross-validation, which significantly enhances the reliability of the findings by reducing the risk of false-positive signals. Furthermore, this multi-method approach allows for more precise characterization of AE reporting patterns, as each algorithm offers unique statistical strengths that are particularly suited to different types of adverse events. The primary objectives of this study were to characterize core disproportionality reporting signal patterns among BTK inhibitors, identify potential rare or unlabeled AE signals, explore associations between reported AEs and fatal outcomes, and describe temporal distributions of selected AE reports. The findings are intended to generate hypotheses for future clinical research and provide reference for optimizing post-marketing safety monitoring strategies, as well as to inform future updates to drug labeling.

## 2 Methods

### 2.1 Study design and data source

This study employed a retrospective pharmacovigilance design utilizing data from the FAERS, encompassing reports from Q1 2004 to Q1 2025. The raw data were sourced from publicly available ASCII files. The analysis focused on the four BTK inhibitors approved and marketed in the United States: ibrutinib, acalabrutinib, zanubrutinib, and pirtobrutinib, with their respective approval datas summarized in Table 1. For each agent, all AE reports where the drug was designated as the primary suspect (PS) were extracted, covering the period from its individual FDA approval date through Q1 2025. The search strategy incorporated all known generic names, brand names, and associated text terms identified from standard sources. Data from seven core FAERS tables were integrated: DEMO (demographic information), DRUG (medication records), OUTC (outcome information), RPSR (reporting source), THER (treatment information), INDI (indication), and REAC (adverse reactions). Subsequent data processing and statistical analysis were performed using R software (version 4.4.2).

**Table 1:** Basic information of the four BTK inhibitors included in the study.

Drug Name	Brand Name	Manufacturer	Generation	FDA Approval Date
Ibrutinib	Imbruvica®	Johnson & Johnson/AbbVie	First	November 2013
Acalabrutinib	Calquence®	AstraZeneca	Second	October 2017
Zanubrutinib	Brukinsa®	BeiGene	Second	November 2019
Pirtobrutinib	Jaypirca®	Eli Lilly	Third	January 2023

### 2.2 Data screening and cleaning

Data processing involved a sequential protocol comprising three key steps: deduplication, error screening, and standardization. Duplicate reports were identified and removed in accordance with the FDA's FAERS Data Mining Technical Specifications (2021 Edition). Specifically, using the primaryID from the DEMO table as a unique report identifier to link records across all associated tables. A report was classified as a duplicate and excluded if the same primaryID appeared under different caseIDs (unique case identifiers) yet contained identical information for drug names, adverse event types, and outcomes. This procedure ensured systematic deduplication across the entire dataset. Invalid or erroneous entries were excluded based on two predefined criteria. First, drug names were cross-referenced against authoritative sources, including Drugs@FDA, RxNorm, and the European Medicines Agency (EMA) drug database. Records with a drug name matching rate below 80% were regarded as containing spelling errors and were removed. Second, reports were excluded if their adverse event (AE) descriptions could not be mapped to standardized medical terms or if none of the target BTK inhibitors were listed as a suspect drug. Subsequently, all AE descriptions in the retained reports were standardized using the Medical Dictionary for Regulatory Activities (MedDRA), version 27.1 (32, 33). The standardization process involved mapping raw verbatim terms to the corresponding Preferred Terms (PTs), which were then categorized into the appropriate System Organ Classes (SOCs). This two-tiered approach guaranteed terminological consistency and enabled systematic comparison of AEs across all analyzed reports.

### 2.3 Statistical analysis

#### 2.3.1 Descriptive analysis

A descriptive analysis was performed on the included AE reports for the four BTK inhibitors, focusing on patient baseline characteristics and the distribution of AEs. The primary objectives were to elucidate the overall data structure and to identify potential confounding factors. Analyzed demographic characteristics included patient gender, age, and reporting region. For variables with substantial missing data, the missing rate was calculated as follows: (number of missing cases/total number of cases) × 100%. The mechanism of missingness was explored using Little's Missing Completely at Random (MCAR) test; however, given the extremely high proportion of missing demographic information for certain drugs, particularly zanubrutinib, the MCAR assumption could not be reliably confirmed. Multiple imputation using logistic regression for gender and linear regression for agewas therefore performed only as an exploratory approach to assess potential distribution patterns. The imputed datasets were not considered representative of the underlying treated population and were interpreted with caution in subsequent analyses (34, 35). To validate the imputation process, comparative visualization charts were generated to assess the distributions of key variables before and after imputation.

Analysis of AE distribution included an assessment of severity and outcomes. A case classified as serious if it met one or more of the following standard criteria: resulting in death, being life-threatening, causing persistent or significant disability/incapacity, requiring or prolonging hospitalization, resulting in congenital anomaly/birth defect, or constituting another medically important condition. Results were summarized using frequencies and percentages, with particular emphasis placed on describing differences in reported serious cases and fatal outcomes among the four BTK inhibitors. To examine system-level adverse event patterns, the number and proportion of reports for each drug within each SOC were calculated. A heatmap was used to visualize differences in SOC-specific reporting profiles among the inhibitors, facilitating the intuitive identification of high-risk organ systems. For a more detailed analysis, the top 30 most frequently reported PTs for each drug were identified. Reporting proportions and signal strength metrics were calculated, and forest plots were generated to illustrate differences in disproportionality reporting signals among BTK inhibitors.

#### 2.3.2 Safety signal detection

Four internationally recognized pharmacovigilance signal detection algorithms were utilized in this study: the Reporting Odds Ratio (ROR) (36, 37), the Proportional Reporting Ratio (PRR) in conjunction with the Medicines and Healthcare products Regulatory Agency (MHRA) comprehensive criteria (38), the Multi-item Gamma Poisson Shrinker (MGPS) (39), and the Bayesian Confidence Propagation Neural Network (BCPNN) (40). To enhance the robustness and reliability of identified signals, a cross-method validation approach was implemented, whereby a signal was considered substantiated only if detected by multiple algorithms (41, 42). The signal detection analysis was uniformly based on a standard 2 × 2 contingency table for each “drug-AE” combination (Supplementary Table S1). Detailed statistical parameters, decision thresholds, and specific applicability considerations for each methodological approach are systematically summarized in Supplementary Table S2.

### 2.4 Clinical signal prioritization framework

Positive signals associated with BTK inhibitors were systematically evaluated and prioritized using a scoring framework based on four clinically relevant dimensions. The specific scoring criteria, detailed in Table 2 (43), were designed to prioritize detected reporting signals according to their reporting frequency, statistical strength, clinical seriousness, and potential relevance for post-marketing surveillance.

**Table 2:** Criteria and scoring system for prioritizing pharmacovigilance signals of AEs analysis.

Criterium	2 points	1 point	0 point
Reporting rate (cases/non-cases)	>10%	1%–10%	0%–1%
Signal stability (consistency across disproportionality analyses)	3–4 of 4	2 of 4	0–1 of 4
Reported case fatality rate (proportion of reports with death as outcome)	> 50%	25%–50%	< 25%
Clinical relevance (serious likely drug-attributable AEs)	Designated medical event	Important medical event	None

The total score corresponded to one of three priority levels: low (0–2 points), moderate (3–5 points), or high (6–8 points).

### 2.5 Sensitivity analysis

To account for potential follow-up bias arising from differing time-on-market and surveillance periods among the BTK inhibitors, a sensitivity analysis was conducted to evaluate the robustness of key safety signals. This analysis employed a uniform data truncation window, restricting the dataset to AE reports from Q1 2023 to Q1 2025. Signal stability was assessed by comparing signal strengths–specifically, Reporting Odds Ratios (RORs)–between the full observation period (from each drug's FDA approval date through Q1 2025) and the standardized two-year window (Q1 2023–Q1 2025). For the 30 most frequently reported Preferred Terms (PTs) common to all four drugs, the relative change in ROR was quantified using the following formula: ROR difference rate (%) = [(ROR of 2-year window−ROR of full cycle)/ROR of full cycle] × 100%. Signal stability was classified according to predefined criteria: (1) Stable: Absolute ROR difference rate <10% with overlapping 95% confidence intervals (CIs); (2) Basically stable: ROR difference rate of 10%–30% or overlapping 95% CIs; (3) Meaningful change: ROR difference rate >30%; (4) Unstable: Non-overlapping 95% CIs with a change in signal direction. This approach allowed for differentiation between drug-specific risks signals and those potentially influenced by variable follow-up time, thereby evaluating the robustness of the observed reporting signal patterns.

### 2.6 Subgroup analyses

To evaluate the robustness of key safety signals across clinically relevant populations, this study performed subgroup analyses. The analyses defined two subgroups based on reporting characteristics: reports of serious adverse events and reports involving first-line treatment patients. For ibrutinib, acalabrutinib, and zanubrutinib, independent analyses were conducted for both of these subgroups. In contrast, because pirtobrutinib is not globally approved for first-line treatment and the number of related reports was limited, this study only performed an analysis of its overall reports. Within each subgroup, the Reporting Odds Ratio along with its 95% confidence interval served as the core metric to recalculate the strength of each adverse reaction signal. Consistency was primarily assessed by comparing signal performance across different subgroups. This assessment examined the stability of signal direction, meaning whether a signal significant in the overall group remained significant in the subgroups. It also evaluated the persistence of core signal patterns, assessing whether the ranking of characteristic high-risk signals for each drug remained stable. Furthermore, it considered the clinical rationale for changes in signal strength. For example, it assessed whether signals directly associated with serious outcomes such as death showed an expected increase in strength within the serious adverse event subgroup.

### 2.7 Negative control analysis

To assess the specificity of the signal detection method and to examine whether the identified positive signals might stem from widespread reporting bias or structural artifacts within the database, we introduced a negative control analysis. We selected five adverse events with no established pathophysiological link to the mechanism of action of BTK inhibitors or their indications as negative control outcomes, including: osteoarthritis, acne, hypoacusis, tinnitus, and diverticulitis. For these events, a statistically significant positive disproportionality signal was defined as a ROR with a 95% CI lower bound >1.

### 2.8 Temporal distribution analysis of reported adverse events

A descriptive temporal analysis was performed to characterize the distribution of reported AE onset intervals among BTK inhibitors. The observation period was stratified into eight key intervals: 0–30, 31–60, 61–90, 91–120, 121–150, 151–180, 181–360, and >360 days, based on the reported time from drug initiation to AE onset. The distribution of core AE reports across these intervals was visualized using a heatmap to describe early-concentrated and late-concentrated reporting patterns. Subsequently, a 2 × 2 contingency table was constructed to compare fatal and non-fatal cases. Association between reported adverse events and fatal outcomes within the FAERS dataset were descriptively explored using Pearson's chi-square test (44). A nominal *p*-value < 0.05 was considered suggestive of statistical significance in individual tests. To mitigate the risk of false-positive findings resulting from multiple hypothesis testing, two correction methods were applied. First, the Benjamini-Hochberg (BH) procedure (45) was employed to control the false discovery rate (FDR), ensuring that the expected proportion of false positives among all significant results did not exceed 5%. Under this method, an adjusted *p*-value of < 0.05 was considered statistically significant. Second, the Bonferroni correction (46) was utilized to control the family-wise error rate (FWER), restricting the probability of any false positives occurring across the entire set of comparisons to a threshold of 5% or less. This stringent approach was specifically adopted for the rigorous validation of high-risk AEs.

## 3 Results

### 3.1 Study population characteristics

#### 3.1.1 Data screening

**Figure 1:**
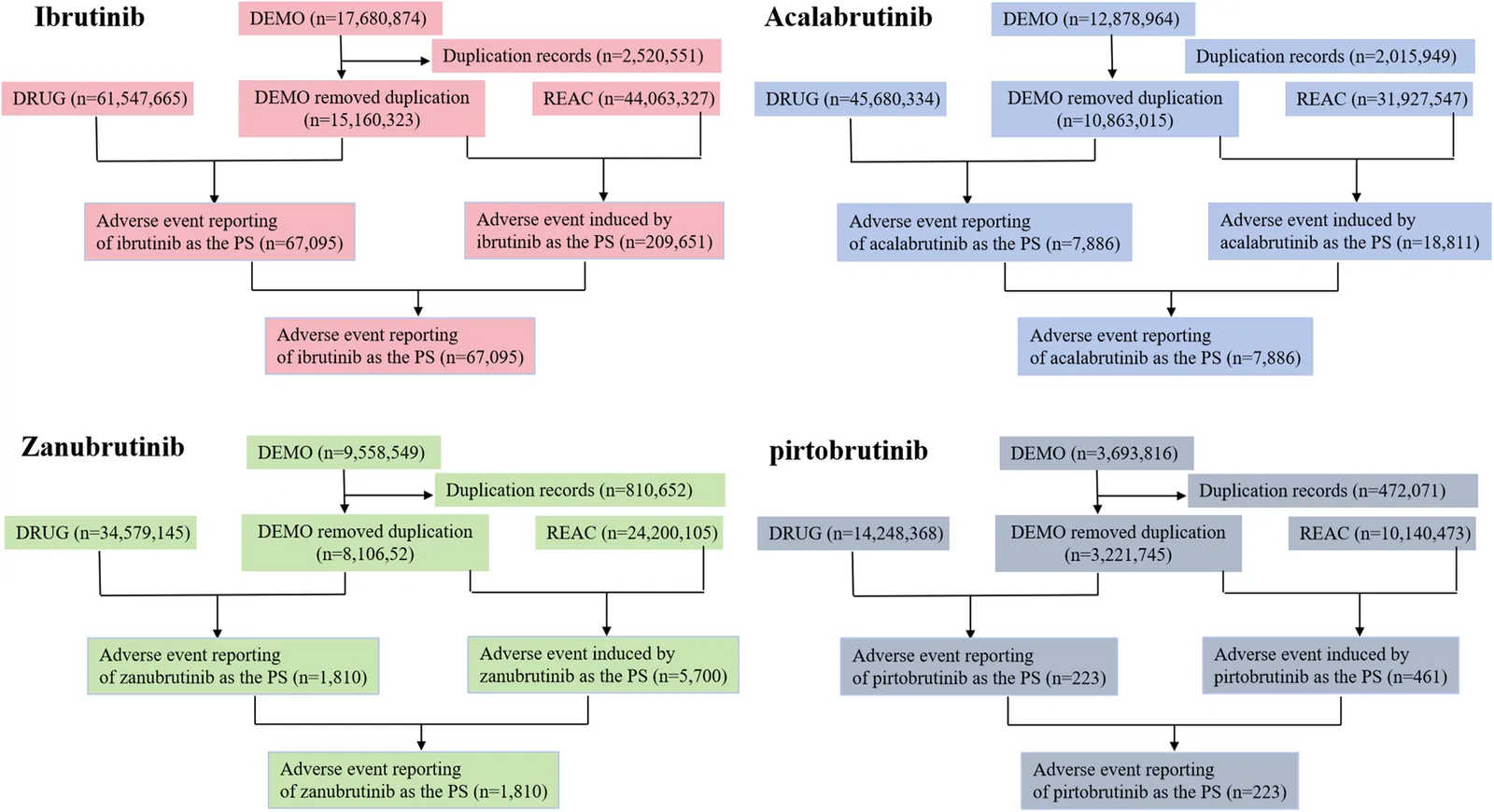
Screening flowchart of adverse event reports for four FDA-approved BTK inhibitors in the FAERS database. DRUG, number of drug records; DEMO, number of demographic records; REAC, number of reaction records; PS, number of adverse event reports with the drug as the primary suspect drug.

This study conducted a systematic screening of adverse event (AE) reports from the FAERS database for four BTK inhibitors: ibrutinib, acalabrutinib, zanubrutinib, and pirtobrutinib. The screening workflow is illustrated in Figure 1. A total of 77,014 AE reports associated with these agents were included in the final analysis. The distribution was as follows: ibrutinib (67,095 cases; 88.2%), acalabrutinib (7,886 cases; 10.4%), zanubrutinib (1,810 cases; 2.4%), and pirtobrutinib (223 cases; 0.3%). Marked disparities in the volume of AE reports were observed across the four BTK inhibitors, with ibrutinib accounting for the highest proportion and pirtobrutinib the lowest. This variation likely reflects differences in post-marketing exposure duration, clinical utilization, reporting volume, and other factors influencing spontaneous reporting.

#### 3.1.2 Demographic and clinical characteristics

Significant differences in baseline characteristics and reported outcomes were observed among the four BTK inhibitors, as detailed in Table 3. Variations in gender and age distributions were notable across the agents. Zanubrutinib exhibited the highest proportion of missing data for gender (97.2%) and age (91.9%), which were recorded as “Unknown”; this extremely high degree of missingness means that demographic analyses for zanubrutinib have very limited reliability and should be interpreted with extreme caution.

**Table 3:** Demographic and adverse event characteristics of BTK inhibitor-associated adverse event reports in FAERS.

Characteristic	Ibrutinib		Acalabrutinib		Zanubrutinib		Pirtobrutinib	
	*n*	%	*n*	%	*n*	%	*n*	%
Sex								
Male	38,435	57.3	3,448	43.7	34	1.9	117	52.5
Female	24,457	36.5	2,020	25.6	17	0.9	54	24.2
Unknown	4,203	6.3	2,418	30.7	1,759	97.2	52	23.3
Age (years)								
<18	78	0.1	0	0	0	0	0	0
18–64	7,022	10.5	462	5.9	30	1.7	27	12.1
65–85	20,812	31.0	1,831	23.2	103	5.7	83	37.2
>85	3,004	4.5	386	4.9	13	0.7	9	4.0
Unknown	36,179	53.9	5,207	66.0	1,664	91.9	104	46.6
Reporting Country								
United States	49,218	73.4	6,187	78.5	1,084	59.9	172	77.1
Other	13,368	19.9	1,696	21.5	725	40.0	51	22.9
Unknown	4,509	6.7	3	0	1	0.1	0	0
Outcomes								
Congenital Anomaly	3	0	5	0.1	2	0.1	0	0
Death	9,427	14.1	1,945	24.7	140	7.7	66	29.6
Disability	231	0.3	46	0.6	8	0.4	1	0.4
Hospitalization	18,117	27.0	1,575	20.0	464	25.6	39	17.5
Life-Threatening	557	0.8	105	1.3	21	1.2	4	1.8
Other	38,760	57.8	4,210	53.4	1,175	64.9	113	50.7
Serious cases								
No	16,999	25.3	1,461	18.5	571	31.5	83	37.2
Yes	50,096	74.7	6,425	81.5	1,239	68.5	140	62.8

The United States was the primary source of AE reports for all four BTK inhibitors. Over 70% of reports for ibrutinib, acalabrutinib, and pirtobrutinib originated from the U.S., while the proportion for zanubrutinib was lower, at 59.9%. Differences were also observed in AE outcomes: the reporting proportions for fatal events were significantly higher for acalabrutinib (24.7%) and pirtobrutinib (29.6%) than for ibrutinib (14.1%) and zanubrutinib (7.7%). In terms of hospitalization events, ibrutinib (27.0%) and zanubrutinib (25.6%) showed relatively higher reporting proportions. The proportion of serious cases exceeded 50% for all agents, with acalabrutinib having the highest proportion (81.5%), followed by ibrutinib (74.7%), zanubrutinib (68.5%), and pirtobrutinib (62.8%), indicating differences in the distribution of reported fatal and serious outcomes among FAERS reports for these agents.

Supplementary Figure S1 illustrates the annual trends in AE reports for the four BTK inhibitors from 2013 to 2025. As the first marketed agent, ibrutinib showed a continuous increase in AE reports, peaking in 2022, followed by a gradual decline. AE reports for the other BTK inhibitors continued to rise through the study period, though the count for 2025 is lower due to data availability only through Q1.

#### 3.1.3 Data completeness assessment

This study evaluated the completeness of core variables in BTK inhibitor-related AE reports from the FAERS database and assessed the feasibility of multiple imputation for handling the extensive missing data in zanubrutinib reports. Data completeness was quantified following STROBE guidelines (47), with results summarized in Supplementary Table S3. Little's Missing Completely at Random (MCAR) test applied to zanubrutinib data yielded a chi-square value of 0 with 0 degrees of freedom and a *p*-value of 1. Given the three distinct missing data patterns observed, these results should not be interpreted as confirming the MCAR assumption because the extremely high proportion of missing demographic data substantially limits the statistical power of the MCAR test. Therefore, multiple imputation was performed only as an exploratory approach. Multiple imputation was subsequently performed for missing gender and age data in zanubrutinib reports. The imputation process included convergence verification, as shown in Supplementary Figure S2, where the iteration plot for age group imputation demonstrated stable means and standard deviations, consistent with original data characteristics and clinical expectations, confirming model convergence. Gender distribution validation, illustrated in Supplementary Figure S3, indicated consistently higher proportions of male patients across 10 imputation rounds, with minimal variation from the original trend, supporting the convergence of the imputation procedure.

After multiple imputation, gender and age distributions for all four BTK inhibitors are presented in Supplementary Table S4 and visualized in stacked bar charts (Supplementary Figure S4 for gender, Supplementary Figure S5 for age). All four BTK inhibitors showed a higher proportion of male patients, with pirtobrutinib having the highest male proportion (76.2%), followed by zanubrutinib (73.1%), acalabrutinib (63.1%), and ibrutinib (61.8%). Regarding age distribution, the 65–85 years group was predominant, accounting for over 60% of reports for ibrutinib (73.9%), zanubrutinib (66.7%), acalabrutinib (62.0%) and pirtobrutinib (64.6%). Notably, acalabrutinib showed a higher proportion of reports in the > 85 years group (20.4%), highlighting potential medication safety considerations in the elderly population.

### 3.2 Signal detection characteristics at the SOC level

We conducted a disproportionality analysis using multiple algorithms (including the ROR, PRR with MHRA criteria, EBGM, and BCPNN) to identify AE signals at the SOC level. Supplementary Table S5 summarizes the number of positive signal reports and quantitative metrics for the four BTK inhibitors, revealing distinct disproportionality signal patterns of AEs.

**Figure 2:**
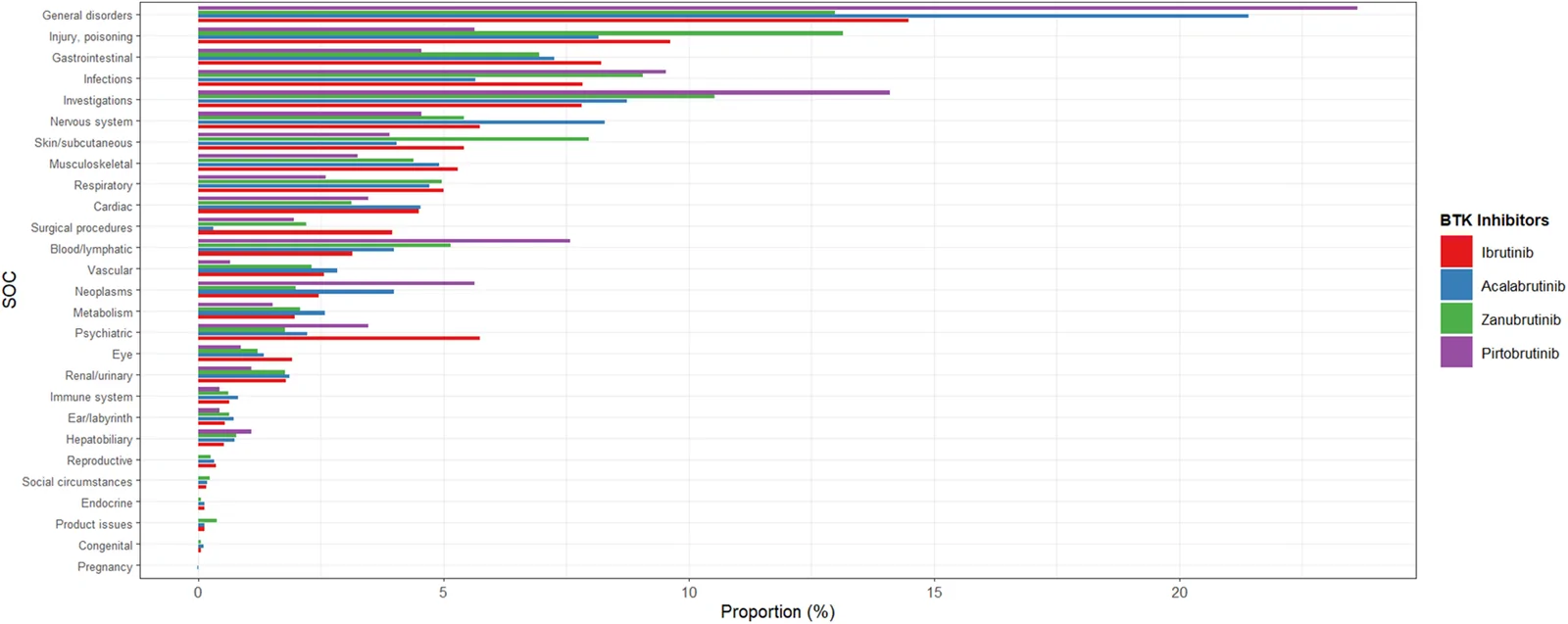
Adverse event distribution by SOC across FDA-approved BTK inhibitors.

**Figure 3:**
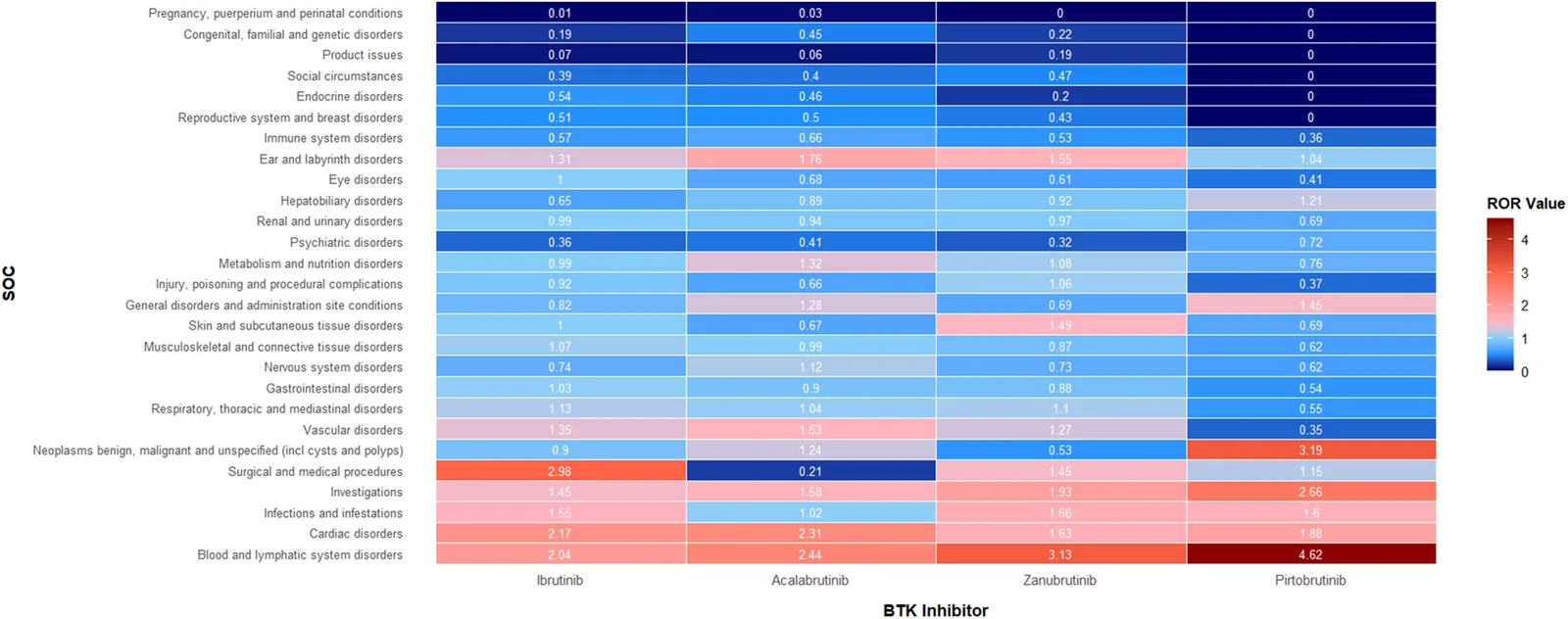
ROR heatmap by SOC for four FDA-approved BTK inhibitors. Horizontal axis: BTK inhibitor type (Ibrutinib, Acalabrutinib, Zanubrutinib, Pirtobrutinib). Vertical axis: System Organ Class (SOC) categories. Color gradient: Red = ROR > 1 (higher disproportionality); Blue = ROR < 1 (lower disproportionality); ROR = 1 is the cut off. ROR values displayed with 95% confidence intervals (95% CI) in table annotations.

Figure 2 illustrates the proportional distribution of AE reports across SOCs for the four BTK inhibitors. The ROR-based heatmap in Figure 3 provides a visual comparison of SOC-level disproportionality reporting signal patterns among the four BTK inhibitors. For ibrutinib, the most frequently reported SOCs were General disorders and administration site conditions and Injury, poisoning and procedural complications, though both exhibited ROR < 1, indicating weak reporting associations. Stronger signals were observed for Cardiac disorders, Surgical and medical procedures, Blood and lymphatic system disorders, and Infections and infestations. Acalabrutinib showed the highest report count in General disorders and administration site conditions, with a positive association. Elevated disproportionality reporting signals were also observed for Cardiac disorders, Blood and lymphatic system disorders, Vascular disorders, and Ear and labyrinth disorders. Zanubrutinib demonstrated a pronounced signal for Skin and subcutaneous tissue disorders and, among the four agents, the highest disproportionality for Infections and infestations. Although pirtobrutinib had a lower overall report volume, its disproportionality metrics identified notable reporting signals for Blood and lymphatic system disorders and other SOC categories, along with elevated signals for Neoplasms benign, malignant and unspecified (including cysts and polyps) and Infections and infestations.

Notably, all four BTK inhibitors shared three SOCs warranting clinical attention: Blood and lymphatic system disorders, Cardiac disorders, and Infections and infestations. When ranked by ROR in descending order, the signal strengths were as follows: Blood and lymphatic system disorders: pirtobrutinib (4.62) > zanubrutinib (3.13) > acalabrutinib (2.44) > ibrutinib (2.04); Cardiac disorders: acalabrutinib (2.31) > ibrutinib (2.17) > pirtobrutinib (1.88) > zanubrutinib (1.63); Infections and infestations: zanubrutinib (1.66) > pirtobrutinib (1.60) > ibrutinib (1.55) > acalabrutinib (1.02). Additional key findings include a unique Neoplasm-related risk signal for pirtobrutinib. In contrast, both acalabrutinib and zanubrutinib showed ROR < 1 for Gastrointestinal disorders, suggesting weaker diarrhea-related disproportionality reporting signals compared with ibrutinib. All the above differences reflect disparities in disproportionality reporting signals.

### 3.3 Signal characteristics and clinical priority at the PT level

**Figure 4:**
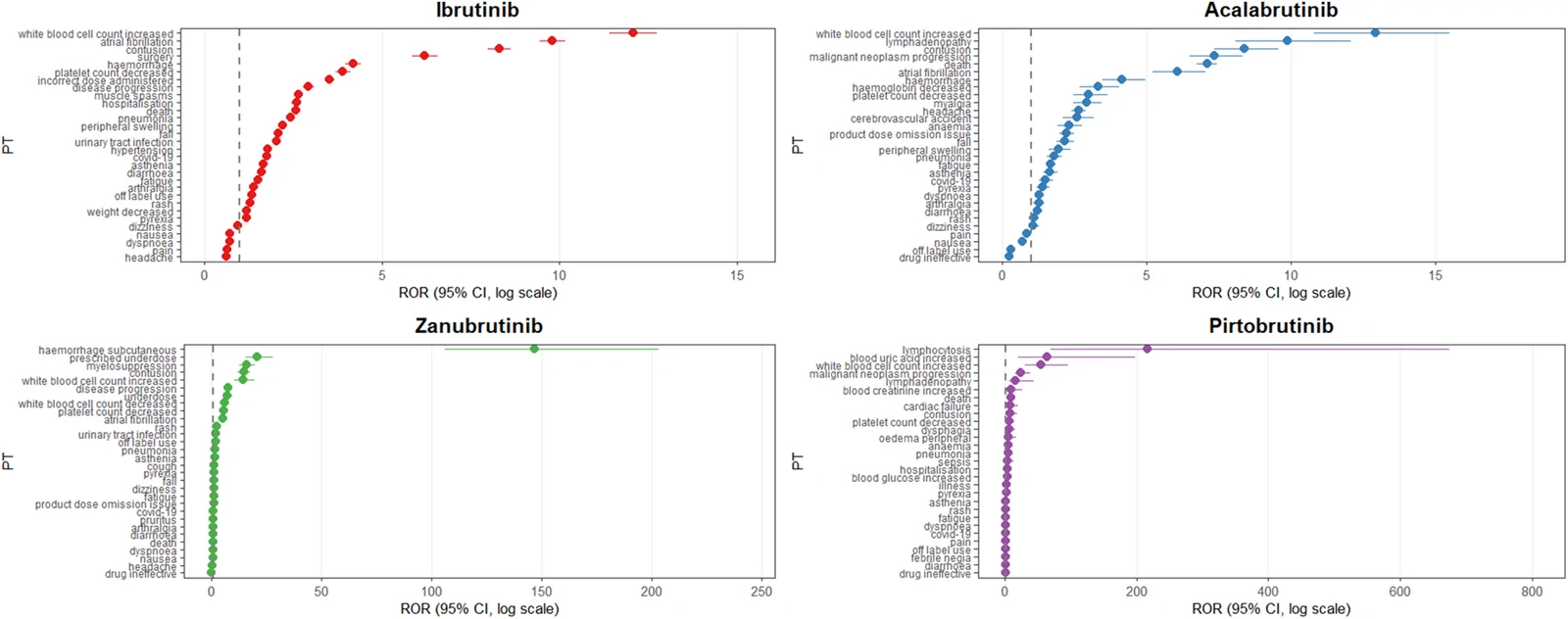
Forest plot of signal detection metrics (ROR with 95% CI) for four FDA-approved BTK inhibitors at the PT level. Horizontal axis: Logarithmic scale of Reporting Odds Ratio (ROR) with 95% confidence interval (95% CI). Vertical axis: Individual Preferred Terms (PTs) ranked by descending ROR magnitude. Color coding: Distinct colors represent different BTK inhibitors, enabling direct comparison of signal strength for PT-drug associations.

This study analyzed the top 30 most frequently reported PTs for each of the four BTK inhibitors. High-priority adverse event reporting signals and key monitoring areas for each agent were identified by integrating signal strength metrics with clinical priority scores, as detailed in Tables 4. Signal strengths at the PT level, ranked by ROR, are visualized in a forest plot (Figure 4).

**Table 4A:** Signal Detection Metrics and Clinical Priority Assessment Criteria for Top 30 PTs by Report Count of Ibrutinib.

No	Ibrutinib	Case Reports	ROR (95% CI)	PRR (*χ*^2^)	EBGM (EBGM_05_)	IC (IC_025_)	Reporting rate	Signal stability	Case fatality rate	Clinical relevance	Total score	clinical prioritization
1	Death	7,642	2.58 (2.52–2.64)	2.52 (7,055.45)	2.51 (2.45)	1.33 (1.29)	1	2	2	1	6	high
2	off label use	4,547	1.34 (1.3–1.38)	1.33 (376.47)	1.33 (1.29)	0.41 (0.37)	1	1	0	0	2	low
3	fatigue	4,120	1.52 (1.47–1.56)	1.51 (704.57)	1.5 (1.46)	0.59 (0.54)	1	1	0	0	2	low
4	diarrhoea	3,574	1.6 (1.55–1.65)	1.59 (786.52)	1.59 (1.53)	0.67 (0.62)	1	1	0	0	2	low
5	atrial fibrillation	2,941	9.8 (9.44–10.17)	9.68 (21,900.66)	9.29 (8.95)	3.22 (3.16)	1	2	0	1	4	moderate
6	incorrect dose administered	2,713	3.53 (3.4–3.67)	3.5 (4,770.76)	3.45 (3.32)	1.79 (1.73)	1	2	0	0	3	moderate
7	pneumonia	2,651	2.44 (2.35–2.53)	2.42 (2,194.09)	2.4 (2.31)	1.27 (1.21)	1	2	0	1	4	moderate
8	contusion	2,576	8.3 (7.98–8.63)	8.21 (15,713.24)	7.94 (7.63)	2.99 (2.93)	1	2	0	1	4	moderate
9	fall	2,263	2.07 (1.99–2.16)	2.06 (1,228.24)	2.05 (1.97)	1.04 (0.97)	1	2	0	0	3	moderate
10	asthenia	2,060	1.67 (1.6–1.75)	1.67 (548.95)	1.66 (1.59)	0.73 (0.67)	0	1	0	0	1	low
11	arthralgia	1,968	1.38 (1.32–1.44)	1.37 (199.21)	1.37 (1.31)	0.45 (0.39)	0	1	0	0	1	low
12	nausea	1,894	0.73 (0.7–0.76)	0.73 (190.83)	0.73 (0.7)	−0.45 (−0.52)	0	0	0	0	0	low
13	rash	1,886	1.28 (1.23–1.34)	1.28 (117.04)	1.28 (1.22)	0.36 (0.29)	0	1	0	0	1	low
14	muscle spasms	1,624	2.65 (2.52–2.78)	2.63 (1,629.39)	2.61 (2.49)	1.39 (1.31)	0	2	0	0	2	low
15	dizziness	1,529	0.94 (0.89–0.99)	0.94 (5.86)	0.94 (0.89)	−0.09 (−0.16)	0	0	0	0	0	low
16	hospitalisation	1,512	2.59 (2.46–2.72)	2.57 (1,442.02)	2.56 (2.43)	1.35 (1.28)	0	2	0	0	2	low
17	haemorrhage	1,437	4.18 (3.97–4.41)	4.16 (3,391.07)	4.1 (3.89)	2.04 (1.96)	0	2	0	2	2	low
18	pain	1,422	0.65 (0.62–0.68)	0.65 (268.32)	0.65 (0.62)	−0.62 (−0.69)	0	0	0	0	0	low
19	peripheral swelling	1,410	2.2 (2.09–2.32)	2.19 (910.06)	2.18 (2.07)	1.13 (1.05)	0	2	0	0	2	low
20	platelet count decreased	1,366	3.89 (3.69–4.11)	3.87 (2,862.49)	3.82 (3.62)	1.93 (1.85)	0	2	0	0	2	low
21	covid-19	1,360	1.75 (1.66–1.85)	1.75 (432.63)	1.74 (1.65)	0.8 (0.72)	0	1	0	0	1	low
22	dyspnoea	1,349	0.71 (0.68–0.75)	0.71 (154.25)	0.72 (0.68)	−0.48 (−0.56)	0	0	0	0	0	low
23	pyrexia	1,332	1.2 (1.14–1.27)	1.2 (43.59)	1.2 (1.13)	0.26 (0.18)	0	1	0	0	1	low
24	white blood cell count increased	1,321	12.06 (11.41–12.75)	11.99 (12,597.35)	11.4 (10.78)	3.51 (3.42)	0	2	0	0	2	low
25	headache	1,298	0.61 (0.57–0.64)	0.61 (326.7)	0.61 (0.58)	−0.71 (−0.79)	0	0	0	0	0	low
26	hypertension	1,206	1.78 (1.68–1.88)	1.77 (403.94)	1.77 (1.67)	0.82 (0.74)	0	1	0	0	1	low
27	urinary tract infection	1,183	2.03 (1.92–2.15)	2.03 (609.69)	2.02 (1.9)	1.01 (0.93)	0	2	0	0	2	low
28	disease progression	1,176	2.93 (2.77–3.11)	2.92 (1,470.81)	2.9 (2.73)	1.53 (1.45)	0	2	0	0	2	low
29	surgery	1,159	6.19 (5.84–6.57)	6.16 (4,875.31)	6.02 (5.67)	2.59 (2.5)	0	2	0	0	2	low
30	weight decreased	1,125	1.2 (1.13–1.27)	1.2 (37.58)	1.2 (1.13)	0.26 (0.18)	0	1	0	0	1	low

Signal detection metrics: Reporting Odds Ratio (ROR) with 95% confidence interval (95% CI); Proportional Reporting Ratio (PRR) with chi-square statistic (χ²); Empirical Bayes Geometric Mean (EBGM) with 5th percentile (EBGM_05_); Information Component (IC) with 25th percentile (IC_025_). Clinical priority assessment criteria: Report proportion; Signal stability; Case fatality rate; Clinical relevance; Composite score.

**Table 4B:** Signal detection metrics and clinical priority assessment criteria for Top 30 PTs by report count of acalabrutinib.

No	Acalabrutinib	Case Reports	ROR (95% CI)	PRR (χ^2^)	EBGM (EBGM_05_)	IC (IC_025_)	Reporting rate	Signal stability	Case fatality rate	Clinical relevance	Total score	clinical prioritization
1	death	1,766	7.09 (6.75–7.45)	6.52 (8,339.15)	6.5 (6.19)	2.7 (2.62)	2	2	2	1	7	high
2	headache	465	2.66 (2.42–2.91)	2.62 (467.85)	2.61 (2.38)	1.39 (1.25)	1	2	0	0	3	moderate
3	fatigue	404	1.69 (1.53–1.87)	1.68 (111.26)	1.67 (1.52)	0.74 (0.6)	1	1	0	0	2	low
4	product dose omission issue	281	2.23 (1.98–2.51)	2.21 (186.85)	2.21 (1.96)	1.14 (0.96)	1	2	0	0	3	moderate
5	malignant neoplasm progression	255	7.34 (6.49–8.31)	7.26 (1,372.32)	7.23 (6.39)	2.85 (2.64)	1	2	0	1	4	moderate
6	diarrhoea	246	1.23 (1.09–1.4)	1.23 (10.72)	1.23 (1.08)	0.3 (0.11)	1	1	0	0	2	low
7	contusion	223	8.38 (7.34–9.57)	8.29 (1,425.13)	8.26 (7.23)	3.05 (2.81)	1	2	0	0	3	moderate
8	dyspnoea	207	1.28 (1.12–1.47)	1.28 (12.71)	1.28 (1.12)	0.36 (0.15)	1	1	0	0	2	low
9	fall	206	2.18 (1.9–2.51)	2.17 (130.69)	2.17 (1.89)	1.12 (0.91)	1	2	0	0	3	moderate
10	asthenia	174	1.66 (1.43–1.92)	1.65 (44.81)	1.65 (1.42)	0.72 (0.5)	0	1	0	0	1	low
11	pneumonia	174	1.79 (1.55–2.08)	1.79 (60.55)	1.79 (1.54)	0.84 (0.61)	0	1	0	1	2	low
12	pain	171	0.86 (0.74–0.99)	0.86 (4.13)	0.86 (0.74)	−0.22 (−0.44)	0	0	0	0	0	low
13	atrial fibrillation	169	6.06 (5.21–7.05)	6.02 (705.41)	6 (5.15)	2.58 (2.32)	0	2	0	1	3	moderate
14	arthralgia	161	1.27 (1.08–1.48)	1.26 (8.88)	1.26 (1.08)	0.34 (0.11)	0	1	0	0	1	low
15	nausea	153	0.7 (0.59–0.82)	0.7 (20)	0.7 (0.6)	−0.52 (−0.75)	0	0	0	0	0	low
16	rash	147	1.1 (0.93–1.29)	1.1 (1.26)	1.1 (0.93)	0.13 (−0.11)	0	0	0	0	0	low
17	dizziness	146	1.08 (0.92–1.28)	1.08 (0.94)	1.08 (0.92)	0.12 (−0.12)	0	0	0	0	0	low
18	covid-19	146	1.51 (1.29–1.78)	1.51 (25.24)	1.51 (1.28)	0.59 (0.35)	0	1	0	0	1	low
19	pyrexia	136	1.4 (1.18–1.65)	1.39 (15.14)	1.39 (1.18)	0.48 (0.23)	0	1	0	0	1	low
20	myalgia	132	2.92 (2.46–3.46)	2.9 (164.63)	2.9 (2.44)	1.54 (1.26)	0	2	0	0	2	low
21	haemorrhage	121	4.15 (3.47–4.97)	4.13 (287.08)	4.13 (3.45)	2.04 (1.75)	0	2	0	0	2	low
22	white blood cell count increased	121	12.93 (10.8–15.47)	12.85 (1,313.06)	12.76 (10.67)	3.67 (3.28)	0	2	0	0	2	low
23	anaemia	119	2.32 (1.93–2.78)	2.31 (88.4)	2.31 (1.93)	1.21 (0.93)	0	2	0	0	2	low
24	peripheral swelling	111	1.96 (1.63–2.37)	1.96 (52.17)	1.96 (1.62)	0.97 (0.68)	0	1	0	0	1	low
25	drug ineffective	110	0.25 (0.21–0.3)	0.25 (245.83)	0.25 (0.21)	−1.97 (−2.24)	0	0	0	0	0	low
26	off label use	108	0.3 (0.25–0.37)	0.31 (171.54)	0.31 (0.25)	−1.7 (−1.97)	0	0	0	0	0	low
27	platelet count decreased	97	3 (2.46–3.67)	2.99 (128.86)	2.99 (2.45)	1.58 (1.26)	0	2	0	0	2	low
28	lymphadenopathy	97	9.88 (8.09–12.07)	9.84 (765.99)	9.79 (8.01)	3.29 (2.87)	0	2	0	0	2	low
29	cerebrovascular accident	95	2.6 (2.12–3.18)	2.59 (92.78)	2.59 (2.11)	1.37 (1.05)	0	2	0	1	3	moderate
30	haemoglobin decreased	91	3.31 (2.69–4.07)	3.3 (145.53)	3.29 (2.68)	1.72 (1.38)	0	2	0	0	2	low

Signal detection metrics: Reporting Odds Ratio (ROR) with 95% confidence interval (95% CI); Proportional Reporting Ratio (PRR) with chi-square statistic (χ²); Empirical Bayes Geometric Mean (EBGM) with 5th percentile (EBGM_05_); Information Component (IC) with 25th percentile (IC_025_). Clinical priority assessment criteria: Report proportion; Signal stability; Case fatality rate; Clinical relevance; Composite score.

**Table 4C:** Signal detection metrics and clinical priority assessment criteria for Top 30 PTs by report count of zanubrutinib.

No	Zanubrutinib	Case Reports	ROR (95% CI)	PRR (χ^2^)	EBGM (EBGM_05_)	IC (IC_025_)	Reporting rate	Signal stability	Case fatality rate	Clinical relevance	Total score	clinical prioritization
1	off label use	244	2.26 (1.99–2.57)	2.21 (164.87)	2.21 (1.94)	1.14 (0.95)	1	2	0	0	3	moderate
2	contusion	117	14.81 (12.33–17.79)	14.53 (1,471.02)	14.48 (12.06)	3.86 (3.43)	1	2	0	0	3	moderate
3	rash	98	2.46 (2.02–3.01)	2.44 (83.51)	2.44 (1.99)	1.28 (0.97)	1	2	0	0	3	moderate
4	fatigue	93	1.29 (1.05–1.58)	1.28 (5.9)	1.28 (1.05)	0.36 (0.06)	1	1	0	0	2	low
5	disease progression	91	7.79 (6.33–9.58)	7.68 (528.77)	7.67 (6.23)	2.94 (2.53)	1	2	0	0	3	moderate
6	myelosuppression	86	16.24 (13.12–20.11)	16.01 (1,207.16)	15.96 (12.89)	4 (3.45)	1	2	0	1	4	moderate
7	death	77	0.95 (0.76–1.19)	0.95 (0.22)	0.95 (0.76)	−0.08 (−0.41)	1	0	0	1	2	low
8	white blood cell count decreased	66	6.17 (4.84–7.86)	6.11 (281.89)	6.1 (4.78)	2.61 (2.15)	1	2	0	0	3	moderate
9	product dose omission issue	61	1.2 (0.93–1.55)	1.2 (2.02)	1.2 (0.93)	0.26 (−0.11)	1	0	0	0	1	low
10	diarrhoea	58	0.97 (0.75–1.26)	0.97 (0.05)	0.97 (0.75)	−0.04 (−0.42)	1	0	0	0	1	low
11	platelet count decreased	57	5.73 (4.41–7.44)	5.68 (220.03)	5.68 (4.37)	2.5 (2.01)	1	2	0	0	3	moderate
12	asthenia	52	1.69 (1.28–2.21)	1.68 (14.34)	1.68 (1.28)	0.75 (0.33)	0	1	0	0	1	low
13	dizziness	51	1.3 (0.99–1.72)	1.3 (3.57)	1.3 (0.99)	0.38 (−0.03)	0	0	0	0	0	low
14	nausea	50	0.78 (0.59–1.03)	0.78 (3.14)	0.78 (0.59)	−0.36 (−0.76)	0	0	0	0	0	low
15	pneumonia	49	1.69 (1.28–2.25)	1.69 (13.84)	1.69 (1.27)	0.76 (0.33)	0	1	0	1	2	low
16	prescribed underdose	48	21.1 (15.87–28.05)	20.93 (906.67)	20.83 (15.67)	4.38 (3.48)	0	2	0	0	2	low
17	atrial fibrillation	44	5.23 (3.89–7.04)	5.2 (149.37)	5.2 (3.86)	2.38 (1.82)	0	2	0	1	3	moderate
18	underdose	44	7.33 (5.45–9.87)	7.29 (238.46)	7.28 (5.41)	2.86 (2.24)	0	2	0	0	2	low
19	dyspnoea	43	0.9 (0.67–1.21)	0.9 (0.49)	0.9 (0.67)	−0.15 (−0.59)	0	0	0	0	0	low
20	covid-19	43	1.11 (0.82–1.5)	1.11 (0.49)	1.11 (0.82)	0.15 (−0.29)	0	0	0	0	0	low
21	cough	42	1.54 (1.14–2.09)	1.54 (7.89)	1.54 (1.13)	0.62 (0.16)	0	1	0	0	1	low
22	pyrexia	40	1.36 (0.99–1.85)	1.35 (3.7)	1.35 (0.99)	0.44 (−0.03)	0	0	0	0	0	low
23	white blood cell count increased	40	14.52 (10.64–19.83)	14.43 (498.43)	14.38 (10.53)	3.85 (2.99)	0	2	0	0	2	low
24	arthralgia	40	1 (0.73–1.36)	1 (0)	1 (0.73)	−0.01 (−0.46)	0	0	0	0	0	low
25	pruritus	38	1.05 (0.76–1.45)	1.05 (0.09)	1.05 (0.76)	0.07 (−0.39)	0	0	0	0	0	low
26	haemorrhage subcutaneous	38	146.81 (106.14–203.07)	145.84 (5,284.76)	141.03 (101.95)	7.14 (4.47)	0	2	0	0	2	low
27	fall	37	1.33 (0.97–1.84)	1.33 (3.07)	1.33 (0.96)	0.41 (−0.07)	0	0	0	0	0	low
28	urinary tract infection	36	2.28 (1.64–3.16)	2.27 (25.61)	2.27 (1.63)	1.18 (0.66)	0	2	0	0	2	low
29	drug ineffective	36	0.28 (0.2–0.38)	0.28 (68.29)	0.28 (0.2)	−1.84 (−2.29)	0	0	0	0	0	low
30	headache	36	0.7 (0.5–0.97)	0.7 (4.77)	0.7 (0.5)	−0.52 (−0.98)	0	0	0	0	0	low

Signal detection metrics: Reporting Odds Ratio (ROR) with 95% confidence interval (95% CI); Proportional Reporting Ratio (PRR) with chi-square statistic (χ²); Empirical Bayes Geometric Mean (EBGM) with 5th percentile (EBGM_05_); Information Component (IC) with 25th percentile (IC_025_). Clinical priority assessment criteria: Report proportion; Signal stability; Case fatality rate; Clinical relevance; Composite score.

**Table 4D:** Signal detection metrics and clinical priority assessment criteria for Top 30 PTs by report count of pirtobrutinib.

No	Pirtobrutinib	Case Reports	ROR (95% CI)	PRR (χ^2^)	EBGM (EBGM_05_)	IC (IC_025_)	Reporting rate	Signal stability	Case fatality rate	Clinical relevance	Total score	clinical prioritization
1	death	50	9.09 (6.78–12.19)	8.21 (320.89)	8.21 (6.12)	3.04 (2.42)	2	2	0	1	5	moderate
2	malignant neoplasm progression	19	24.45 (15.45–38.72)	23.49 (409.36)	23.46 (14.82)	4.55 (2.81)	1	2	2	1	6	high
3	white blood cell count increased	12	54.28 (30.58–96.37)	52.9 (609.81)	52.77 (29.72)	5.72 (2.59)	1	2	0	0	3	moderate
4	pneumonia	11	4.53 (2.49–8.23)	4.44 (29.49)	4.44 (2.44)	2.15 (0.94)	1	2	0	1	4	moderate
5	off label use	10	0.92 (0.49–1.72)	0.92 (0.07)	0.92 (0.49)	−0.12 (−0.99)	1	0	0	0	1	low
6	fatigue	9	1.5 (0.78–2.9)	1.49 (1.47)	1.49 (0.77)	0.57 (−0.42)	1	1	0	0	2	low
7	drug ineffective	7	0.74 (0.35–1.56)	0.74 (0.63)	0.74 (0.35)	−0.43 (−1.41)	1	0	0	0	1	low
8	anaemia	6	5.06 (2.26–11.33)	5.01 (19.3)	5.01 (2.24)	2.32 (0.57)	1	2	0	0	3	moderate
9	pyrexia	6	2.49 (1.11–5.58)	2.47 (5.3)	2.47 (1.11)	1.31 (−0.07)	1	2	0	0	3	moderate
10	dyspnoea	5	1.34 (0.55–3.23)	1.34 (0.43)	1.34 (0.55)	0.42 (−0.85)	1	1	0	0	2	low
11	contusion	5	7.44 (3.08–17.96)	7.37 (27.55)	7.37 (3.05)	2.88 (0.65)	1	2	0	0	3	moderate
12	hospitalisation	5	3.42 (1.42–8.27)	3.4 (8.49)	3.4 (1.41)	1.76 (0.09)	1	2	0	0	3	moderate
13	platelet count decreased	5	6.18 (2.56–14.91)	6.12 (21.45)	6.12 (2.53)	2.61 (0.54)	1	1	0	0	2	low
14	illness	5	2.54 (1.05–6.14)	2.53 (4.63)	2.53 (1.05)	1.34 (−0.18)	1	2	1	1	4	moderate
15	rash	5	1.53 (0.64–3.7)	1.53 (0.92)	1.53 (0.63)	0.61 (−0.7)	1	1	0	0	2	low
16	asthenia	4	1.56 (0.58–4.19)	1.56 (0.81)	1.56 (0.58)	0.64 (−0.81)	0	0	0	0	0	low
17	blood creatinine increased	4	9.74 (3.64–26.07)	9.66 (31.08)	9.66 (3.61)	3.27 (0.52)	0	2	0	0	2	low
18	pain	4	0.98 (0.37–2.62)	0.98 (0)	0.98 (0.37)	−0.03 (−1.32)	0	0	0	0	0	low
19	covid-19	4	1.2 (0.45–3.2)	1.2 (0.13)	1.2 (0.45)	0.26 (−1.1)	0	0	0	0	0	low
20	dysphagia	4	6.03 (2.25–16.14)	5.99 (16.64)	5.99 (2.24)	2.58 (0.28)	0	2	0	0	2	low
21	lymphadenopathy	4	16.39 (6.12–43.87)	16.26 (57.26)	16.25 (6.07)	4.02 (0.71)	0	2	1	0	0	moderate
22	diarrhoea	4	0.82 (0.31–2.19)	0.82 (0.16)	0.82 (0.31)	−0.29 (−1.54)	0	0	0	0	0	low
23	cardiac failure	4	7.62 (2.85–20.41)	7.57 (22.82)	7.56 (2.83)	2.92 (0.41)	0	2	1	1	4	moderate
24	lymphocytosis	3	215.64 (68.91–674.79)	214.24 (630.62)	212.19 (67.81)	7.73 (0.52)	0	1	0	0	1	low
25	oedema peripheral	3	5.5 (1.77–17.11)	5.47 (10.96)	5.47 (1.76)	2.45 (−0.08)	0	1	0	0	1	low
26	febrile neutropenia	3	5.36 (1.72–16.68)	5.33 (10.56)	5.33 (1.71)	2.41 (−0.09)	0	1	1	2	4	moderate
27	arthralgia	3	0.85 (0.27–2.63)	0.85 (0.08)	0.85 (0.27)	−0.24 (−1.63)	0	0	0	0	0	low
28	sepsis	3	4.17 (1.34–12.98)	4.15 (7.18)	4.15 (1.33)	2.05 (−0.24)	0	1	2	1	4	moderate
29	blood uric acid increased	3	63.55 (20.39–198.1)	63.15 (182.98)	62.97 (20.2)	5.98 (0.48)	0	2	0	0	1	low
30	blood glucose increased	3	3.18 (1.02–9.9)	3.17 (4.46)	3.17 (1.02)	1.66 (−0.41)	0	1	0	0	1	low

Signal detection metrics: Reporting Odds Ratio (ROR) with 95% confidence interval (95% CI); Proportional Reporting Ratio (PRR) with chi-square statistic (χ²); Empirical Bayes Geometric Mean (EBGM) with 5th percentile (EBGM_05_); Information Component (IC) with 25th percentile (IC_025_). Clinical priority assessment criteria: Report proportion; Signal stability; Case fatality rate; Clinical relevance; Composite score.

#### 3.3.1 Blood and lymphatic system disorders

Within the SOC of Blood and Lymphatic System Disorder—a core safety domain common to all four BTK inhibitors—ibrutinib was most frequently associated with platelet count decreased (*n* = 1,366; ROR = 3.89) and white blood cell count increased (*n* = 1,321; ROR = 12.06). The latter showed the highest ROR among all PTs in this SOC but was assigned a low clinical priority score. For acalabrutinib, the predominant PTs were anaemia (ROR = 2.32; IC = 1.21) and white blood cell count increased (ROR = 12.93; EBGM = 12.76), the latter exhibiting the strongest signal in this drug yet also receiving low clinical priority. Zanubrutinib was characterized by a distinct high-signal PT of myelosuppression (ROR = 16.24; IC = 4.00; clinical priority: moderate), representing its most notable hematological disproportionality reporting signal, along with white blood cell count decreased (ROR = 6.17; EBGM = 6.10; clinical priority: moderate). For pirtobrutinib, several hematological PTs showed higher ROR values compared to the other inhibitors in this dataset. Specifically, white blood cell count increased showed a markedly elevated signal (ROR = 54.28; IC = 5.72) and anaemia was also strongly associated (ROR = 5.06; EBGM = 5.01).

#### 3.3.2 Cardiac disorders

Atrial fibrillation was identified as the core PT for Cardiac Disorders across the four BTK inhibitors, with following agent-specific characteristics. Among ibrutinib-treated patients, atrial fibrillation represented the predominant PT, with 2,941 reported cases (ROR = 9.8; IC = 3.22). It demonstrated the highest ROR among all PTs and was assigned a moderate clinical priority, indicating that atrial fibrillation represented a prominent cardiovascular-related reporting signal for this inhibitor. Acalabrutinib also exhibited atrial fibrillation as a core PT (ROR = 6.06; EBGM = 6.00), with a statistically significant ROR. Zanubrutinib demonstrated a comparatively lower ROR for atrial fibrillation (5.23) than ibrutinib and acalabrutinib. In the case of pirtobrutinib, atrial fibrillation did not rank among the top 30 reported PTs. Only four cases of cardiac failure were documented (ROR = 7.62), suggesting fewer prominent cardiac disproportionality signals within the available FAERS reports for this agent.

#### 3.3.3 Infections and infestations

Pneumonia was identified as the core PT for Infections and Infestations across all four BTK inhibitors, based on its highest report frequency of AE reports and strongest signal intensity. The agent-specific profiles are summarized as follows. For ibrutinib, pneumonia represented the predominant infection-related event with 2,651 reports (ROR = 2.44) and was assigned a moderate clinical priority. Urinary tract infection was also notable with 1,183 reports (ROR = 2.03) though classified as low clinical priority. Both zanubrutinib and acalabrutinib demonstrated lower ROR values for pneumonia (1.69 and 1.79, respectively), with low clinical priority scores, indicating weaker infection-related disproportionality reporting signals in this dataset. In contrast, pirtobrutinib showed a moderate-priority signal for pneumonia (ROR = 4.53; EBGM = 4.44). Although the absolute number of reports was limited, the elevated signal strength suggests a clinically noteworthy infection-related reporting signal requiring further evaluation.

#### 3.3.4 General disorders and administration site conditions

The SOC for General disorders and administration site conditions encompasses PTs including death, fatigue, and asthenia. Among these, death was identified as a high-priority AE due to its clinical severity. The safety profiles of the four BTK inhibitors in this SOC were characterized as follows: Ibrutinib showed death as the core PT, with 7,642 reports (ROR = 2.58; IC = 1.33) and a high clinical priority, reflecting the severity of this reported outcome category within FAERS. Fatigue was also observed as a secondary PT, though with a lower ROR of 1.52 and low clinical priority. Acalabrutinib exhibited an elevated signal for death, with 1,766 reports (ROR = 7.09; EBGM = 6.50) and a clinical priority score of 7, representing the strongest association among the four inhibitors. Asthenia was reported but assigned low clinical priority. In contrast, zanubrutinib was associated with 77 death reports (ROR = 0.95), indicating no statistically significant signal. Pirtobrutinib demonstrated a notably high ROR of 9.09 for death, accompanied by a moderate clinical priority score.

#### 3.3.5 Analysis of PT in remaining SOCs

Pirtobrutinib exhibited a notably elevated signal for the PT malignant neoplasm progression within the SOC Neoplasms benign, malignant and unspecified (including cysts and polyps) (ROR = 24.45), accompanied by a clinical priority score of 6. For acalabrutinib, the PT malignant neoplasm progression showed an ROR of 7.34, while zanubrutinib demonstrated an ROR of 7.79 for disease progression, both classified as moderate priority. In contrast, malignant neoplasm progression was not observed among the top 30 PTs for ibrutinib. Within the SOC Skin and subcutaneous tissue disorders, the principal PTs included contusion and rash. Zanubrutinib demonstrated a particularly strong signal for haemorrhage subcutaneous (ROR = 146.81), highlighting a strong bleeding-related disproportionality reporting signal that may warrant further clinical investigation. In Gastrointestinal disorders, diarrhea was the core PT for the SOC. Ibrutinib presented a higher risk of diarrhea (ROR = 1.60) compared to acalabrutinib (ROR = 1.23) and zanubrutinib (ROR = 0.97), though all three agents were assigned low clinical priority. For the SOC Nervous system disorders, headache emerged as the core PT, with acalabrutinib showing the highest associated risk (ROR = 2.66) and a moderate priority rating.

In summary, the safety profiles of the four BTK inhibitors reflected both shared SOCs and distinct PT-level signals. A comparative overview of SOC- and PT-level signals is provided in Supplementary Table S6, while Supplementary Table S7 enumerates moderate-priority adverse events warranting key clinical monitoring. Specific monitoring priorities include: atrial fibrillation and pneumonia with ibrutinib; fatal outcomes and anemia with acalabrutinib; myelosuppression and haemorrhage subcutaneous with zanubrutinib; and malignant neoplasm progression and hematological abnormalities with pirtobrutinib. These findings may assist post-marketing safety monitoring by highlighting agent-specific reporting signal patterns at the SOC and PT levels. Clinical decisions should integrate individual patient characteristics, guideline recommendations, and evidence from controlled clinical studies.

#### 3.3.6 Sensitivity analysis

To mitigate potential follow-up bias resulting from differing market entry times and surveillance periods among the four BTK inhibitors, a standardized 2-year observation window (Q1 2023 to Q1 2025) was applied for data truncation. Only AE reports falling within this period were included in the sensitivity analysis. This method effectively controlled for bias attributable to unequal drug exposure durations, allowing evaluation of the stability of disproportionality reporting signals under a standardized observation window. The results of this analysis are summarized in Supplementary Table S8 and described in detail below. Ibrutinib exhibited stable signal strength for its primary clinically relevant PTs—including atrial fibrillation, haemorrhage, and increased white blood cell count—across both the full and truncated time windows. Consistency was also observed for most shared PTs, indicating that core cardiovascular-related disproportionality reporting signals associated with ibrutinib remained stable across different observation windows, suggesting robustness of the observed reporting patterns. These findings further validate the reliability of the full-cycle analysis. Similarly, acalabrutinib demonstrated consistent and robust signals for key clinical PTs such as pneumonia, atrial fibrillation, haemorrhage, and anemia in both analytical periods, supporting the stability of its safety profile. Zanubrutinib also showed minimal fluctuation across all its PTs, with critical risk signals—including myelosuppression and haemorrhage subcutaneous—remaining stable throughout, supporting the stability of these observed reporting signals despite differences in surveillance duration.

#### 3.3.7 Consistency in subgroup analyses

Subgroup analyses were conducted to evaluate the robustness of key adverse event signals. The analyses for ibrutinib, acalabrutinib, and zanubrutinib revealed a high degree of consistency in their core signals across the Serious Adverse Events and First-line Treatment patient populations, as shown in Supplementary Table S9. For instance, ibrutinib's signal for atrial fibrillation remained strongly positive and numerically similar across all subgroups [ROR (95% CI): 9.8 (9.44–10.17) overall, 10.47 (10.05–10.89) for serious AEs, and 9.83 (9.44–10.24) for first-line treatment]. Acalabrutinib's signals for death [ROR: 7.09 (6.75–7.45), 8.86 (8.43–9.32), 7.11 (6.77–7.46)] and increased white blood cell count [ROR: 12.93 (10.80–15.47), 11.19 (9.05–13.84), 12.96 (10.83–15.51)] demonstrated exceptionally strong and stable associations in all subgroups. Zanubrutinib's prominent signals for subcutaneous hemorrhage [ROR: 146.81 (106.14–203.07), 178.07 (127.03–249.64), 147.15 (106.38–203.54)] and myelosuppression [ROR: 16.24 (13.12–20.11), 21.52 (17.38–26.66), 16.09 (12.98–19.94)] consistently maintained a high-intensity pattern across subgroups. As expected, signals directly linked to severe outcomes like death and disease progression showed an anticipated increase in strength within the serious AE subgroup. Notably, the signal patterns in first-line treatment patients were highly similar to those in the overall population, suggesting that these reporting patterns were observed among reports involving first-line treatment patients. For pirtobrutinib, a subgroup analysis for first-line treatment was not performed, as it is not globally approved for this indication and the number of reports in this analysis was relatively limited. Its overall and serious AE signals reflect the unique profile associated with its use in later-line treatment populations. All subgroup analyses are descriptive, revealing stable reporting association patterns within the FAERS database and providing a hypothesis-generating foundation for future research.

#### 3.3.8 Specificity assessment via negative control outcomes

To validate the specificity of our disproportionality analysis, we performed the same statistical testing on five pre-specified negative control outcomes. The results are presented in Supplementary Table S10. For none of the four BTK inhibitors did the negative control outcomes show a statistically significant positive disproportionality signal. The lower bounds of the 95% confidence intervals for all RORs were <1, indicating no over-reporting of these unrelated events among patients exposed to BTK inhibitors. It is noteworthy that data for some negative control outcomes were missing for pirtobrutinib, which is consistent with its relatively recent market approval as of the data cutoff and its smaller post-marketing exposure in the FAERS database.

### 3.4 Time-to-onset distribution of AEs

This section examines the temporal distribution patterns of reported key AEs associated with ibrutinib and compares the overall time-to-onset patterns among the four BTK inhibitors. The analysis aims to characterize differences in the timing distribution of reported AEs and provide descriptive information for post-marketing safety surveillance.

#### 3.4.1 Time-to-onset distribution of PT for ibrutinib

**Figure 5:**
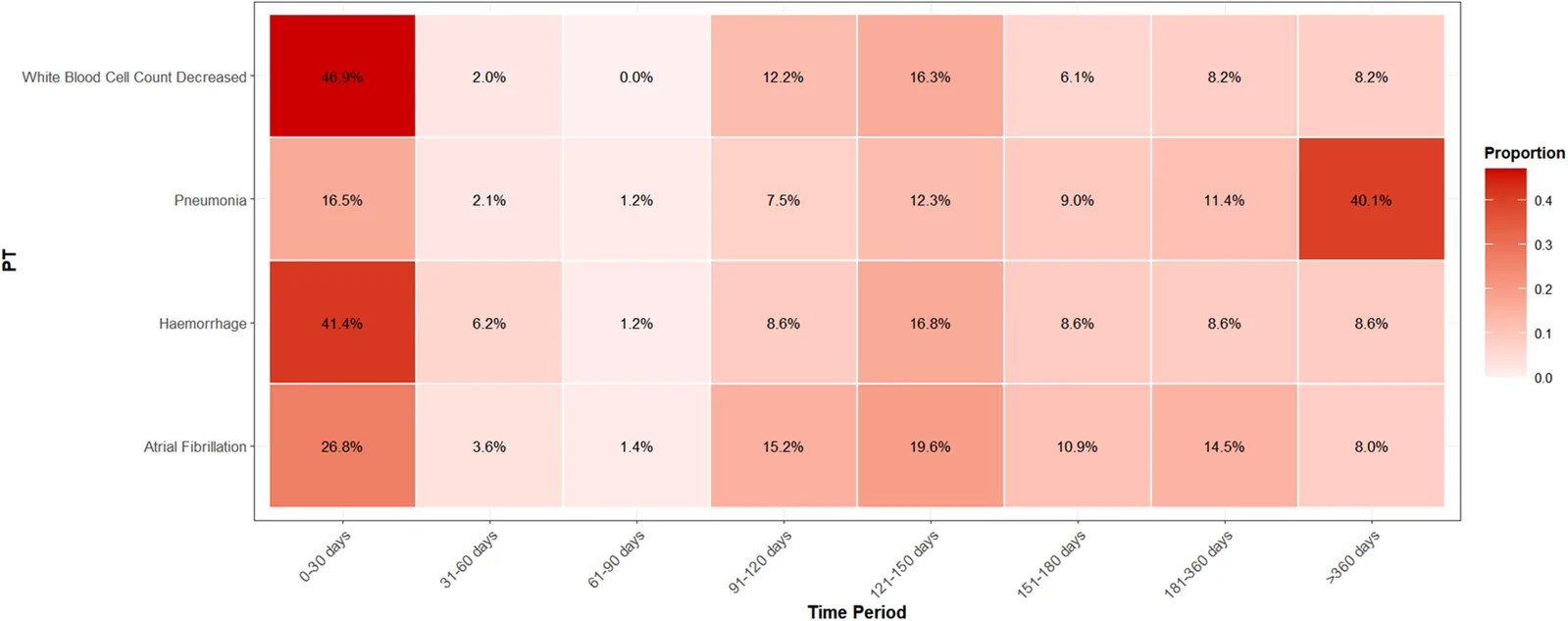
Heatmap of occurrence proportions for Key ibrutinib PTs across time intervals. The heatmap visualizes the distribution of four critical PTs—white blood cell count decreased, pneumonia, haemorrhage, and atrial fibrillation—across eight time intervals (0–30 to >360 days). Color intensity is positively correlated with occurrence proportion, with darker shades represent higher proportions in respective intervals.

Significant heterogeneity was observed in the reported time-to-onset distribution patterns of ibrutinib-associated PTs (Figure 5). For white blood cell count decreased, nearly 50% of reported cases occurred within the first 30 days after treatment initiation, followed by a lower proportion of reports in subsequent intervals, indicating an early-concentrated reporting pattern. In contrast, pneumonia showed a relatively dispersed temporal distribution, with 16.5% of reported cases occurring within the 0–30 day interval and the highest proportion observed in the >360-day interval. This pattern reflects a greater proportion of pneumonia reports among cases with longer reported treatment durations in the FAERS database. Haemorrhage exhibited the highest proportion of reported cases within the first 30 days (41.4%), followed by lower proportions in subsequent intervals (6.2% during 31–60 days and 1.2% during 61–90 days), consistent with an early-concentrated reporting pattern. Atrial fibrillation showed a relatively heterogeneous distribution, with higher proportions of reports observed during the 0–30 days (26.8%), 121–150 days (19.6%), and 181–360 days (14.5%) intervals, without a clear predominance toward either earlier or later reported onset intervals.

#### 3.4.2 Differences in AE onset time trends among the four BTK inhibitors

Supplementary Figure S6 illustrates differences in the reported onset time distribution patterns among the four BTK inhibitors. Pirtobrutinib demonstrated a notably higher proportion of AEs within the first 30 days after treatment initiation compared with the other three BTK inhibitors, followed by a decline in subsequent intervals, representing a relatively early-concentrated reporting pattern. In contrast, ibrutinib showed a higher proportion of AEs in the >360 day interval, reflecting a greater proportion of reports among cases with longer reported treatment durations. Acalabrutinib and zanubrutinib displayed relatively moderate fluctuations in reported AE proportions across different time intervals, without prominent early- or late-concentrated reporting patterns. Overall, these findings indicate differences in the temporal distribution patterns of reported adverse events among BTK inhibitors, rather than differences in time-dependent clinical risk.

### 3.5 Association between AEs and fatal outcomes

This section analyzes the associations between reported AEs and reported fatal outcomes for ibrutinib and acalabrutinib. This analysis is based solely on reported case data and cannot estimate actual mortality rates, survival outcomes, or comparative mortality risks between agents. The evaluation integrates three components: frequency summaries of PTs among reported fatal and non-fatal cases, and visualization of statistical associations between PTs and reported fatal outcomes. Zanubrutinib was not included in detailed comparative analysis because no statistically significant association between reported AEs and fatal outcomes was identified in this dataset (ROR = 0.95, 95% CI: 0.76–1.19). Similarly, pirtobrutinib was not included in subsequent comparisons; although a relatively high proportion of reported fatal outcomes was observed, the limited total sample size (only 50 death-related reports) constrained the robustness of PT-death association signals.

**Figure 6:**
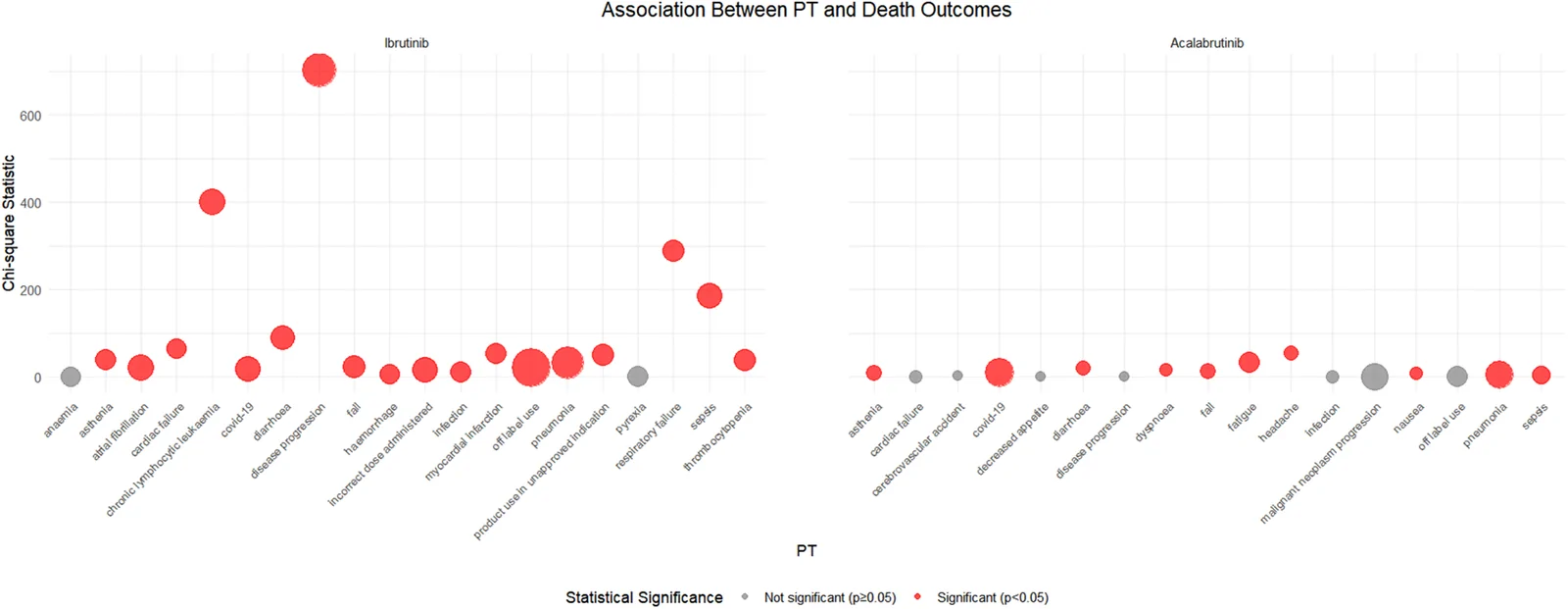
Bubble plot visualizing the associations between PTs and death outcomes. This bubble plot illustrates the association characteristics between PTs and death outcomes for ibrutinib and acalabrutinib. The horizontal axis displays PT names, and the vertical axis shows the chi-square statistic, which quantifies the strength of the frequency difference between death and non-death groups (higher values indicate stronger associations). Bubble color indicates statistical significance: red for *p* < 0.05 (significant) and gray for *p* ≥ 0.05 (non-significant). Bubble size is proportional to the number of death cases associated with each PT, reflecting its prevalence in fatal outcomes.

Differences were observed in the profiles of associations between reported AEs and fatal outcomes between the two inhibitors (Supplementary Table S11 and Figure 6). Ibrutinib showed strong, statistically significant associations between multiple PTs and death. Disease progression, respiratory failure, and chronic lymphocytic leukemia demonstrated the highest chi-square values, indicating stronger statistical associations with reported fatal outcomes. Significant associations were also noted for sepsis, diarrhea, and cardiac failure. In contrast, acalabrutinib exhibited statistically significant associations for only a limited set of PTs, such as headache, dyspnea, and diarrhea. Overall chi-square values for acalabrutinib were considerably lower than those for ibrutinib, reflecting weaker statistical associations between reported AEs and reported fatal outcomes in this dataset.

## 4 Discussion

### 4.1 Principal findings

This study conducted a systematic pharmacovigilance analysis utilizing the FAERS database, providing a comprehensive characterization of disproportionality reporting signals among four marketed BTK inhibitors—ibrutinib, acalabrutinib, zanubrutinib, and pirtobrutinib—through a multi-method signal detection framework. Based on 77,014 real-world AE reports, we applied four internationally recognized algorithms—ROR, PRR (with MHRA criteria), MGPS and BCPNN—to perform cross-validated signal detection at both the SOC and PT levels. This methodological framework was further complemented by a clinical priority scoring system integrating reporting rate, signal consistency, case fatality rate, and clinical relevance. To address potential time-on-market reporting bias, standardized sensitivity analyses were conducted, together with subgroup analyses and negative control testing. Temporal analyses were also performed to characterize AE onset distribution patterns and their associations with reported fatal outcomes. Through this integrated approach, the study describes significant differences in AE reporting signal patterns across BTK inhibitor generations, reveals agent-specific report signal characteristics, and provides a hypothesis-generating foundation for post-marketing safety surveillance and future clinical investigations.

The principal finding of this study demonstrates that while BTK inhibitors share common disproportionality signal domains across generations, they exhibit distinct drug-specific signals at the PT level. Baseline characteristics indicated that AE reports for all four BTK inhibitors predominantly involved male and elderly patients. At the SOC level, three domains—Blood and Lymphatic System Disorders, Cardiac Disorders, and Infections and Infestations—emerged as shared areas of elevated reporting signals. In contrast, substantial divergence in disproportionality signal patterns was observed at the PT level across generations: First-generation ibrutinib was characterized by a strong cardiovascular-related disproportionality reporting signal, particularly for atrial fibrillation (ROR = 9.8). Second-generation inhibitors (acalabrutinib and zanubrutinib) demonstrated weaker atrial fibrillation-related disproportionality signals, with RORs of 6.06 and 5.23, respectively. Zanubrutinib exhibited unique signals for myelosuppression and haemorrhage subcutaneous. Third-generation pirtobrutinib, despite limited data volume, showed pronounced hematological toxicity signals, including increased white blood cell count (ROR = 54.28), along with a significant reporting association with tumor progression. All comparative descriptions above are based on disproportionality signal strength in the FAERS database and do not represent confirmed differences in actual clinical safety risk.

### 4.2 Interpretation of key findings and clinical implications

#### 4.2.1 Baseline characteristics

AE reports for all four BTK inhibitors predominantly involved male patients, who comprised between 61.8% and 76.2% of reported cases. This gender distribution likely reflects the epidemiological characteristics of NHL—the primary indication for BTK inhibitors—rather than indicating gender-specific drug toxicity. NHL incidence is approximately 30% higher in males than in females (48), a difference potentially attributable to anatomical and physiological factors (49). The majority of AE reports across all four drugs originated from elderly patients aged 65–85 years, consistent with the peak incidence of NHL (50) and the widespread clinical application of BTK inhibitors in this demographic. However, age-related physiological changes in elderly patients directly influence medication safety, highlighting two key considerations. First, impaired drug metabolism may elevate risk; ibrutinib is primarily metabolized by CYP3A4 (51) and concomitant liver impairment in elderly patients could contribute to the elevated long-term infection risk observed in this study. Second, the complexity of comorbidity and polypharmacy presents another significant challenge. Elderly patients frequently receive concomitant medications such as anticoagulants, antihypertensives, and hypoglycemic agents. Consequently, individualized evaluation of concomitant medications remains important when interpreting safety signals in elderly patients receiving BTK inhibitors.

Zanubrutinib exhibited substantially higher rates of missing data for gender and age variables compared to the other three BTK inhibitors. Although Little's MCAR test supported the randomness of the missing data and multiple imputation was applied, the inherent limitations of this methodological approach should be acknowledged. Therefore, all demographic-related analyses for zanubrutinib are exploratory only.

Based on the characterized baseline profile of the treated population, this study established a three-dimensional analytical framework using real-world data from the FAERS. This framework encompasses validation and supplementation, comparative differentiation, and clinical translation. It is designed not only to corroborate the robustness of safety signals identified in key randomized controlled trials, within broader and more heterogeneous populations, but also to detect rare and long-term risks. By employing multi-algorithm cross-validation, the framework enables a quantitative comparison of risk disparities among the four BTK inhibitors. The ultimate aim of this analysis is to provide evidence supporting post-marketing safety surveillance and hypothesis generation for future comparative effectiveness and safety studies. Guided by this framework, the subsequent analysis will begin with a systematic examination of blood and lymphatic system disorders. This system organ class is most directly associated with the core physiological function of the BTK signaling pathway.

#### 4.2.2 Blood and lymphatic system disorders

Blood and lymphatic system disorders represent a frequently reported class of AEs across BTK inhibitors, with all four agents demonstrating significant signals at the SOC level, indicating that hematological adverse event reporting signals represent a shared class-related pattern. However, analysis revealed distinct agent-specific disproportionality signal patterns of AEs: ibrutinib was associated with significant signals for platelet count decreased and white blood cell count increased, though these were assigned low clinical priority. The observed increase in white blood cell count, primarily reflecting lymphocytosis, can be explained by an immunologically plausible mechanism. When BTK is effectively inhibited, it interferes with the CXCR4-mediated signaling pathway. The CXCR4 receptor and its ligand CXCL12 form a ke*y* axis responsible for retaining B-cells within protective niche microenvironments in the bone marrow, lymph nodes, and spleen. BTK inhibitors disrupt this axis, reducing the adhesion of malignant B-cells to their stromal niches and promoting their egress from lymphoid tissues and bone marrow reservoirs into the peripheral blood. This redistribution mechanism underlies the pronounced elevation in peripheral blood lymphocyte counts seen following BTK inhibition (52, 53). Notably, nearly half of the reported white blood cell count decreased cases occurred within the first 30 days of treatment initiation, indicating an early-concentrated reporting pattern.

Acalabrutinib exhibited generally mild hematological toxicity, with significant signals limited to anemia and increased white blood cell count. Zanubrutinib demonstrated the strongest myelosuppression-related disproportionality signal among the four inhibitors, suggesting a distinct hematological reporting pattern requiring further evaluation. This clinical observation appears to contrast with *in vitro* data showing that zanubrutinib (1 μM) inhibited CFU-GM formation by only 15%, compared to 42% for ibrutinib, indicating a milder myelosuppressive potential (54). The ALPINE trial reported grade ≥3 neutropenia in 36% of patients but only 2.8% febrile neutropenia (55), complements this trial data by quantifying the relative reporting strength compared to other BTK inhibitors in a real-world context, suggesting that while manageable, it remains a defining feature of its profile. The mechanism of BTK inhibitor-induced hematological toxicity involves dual pathways: direct induction of B-cell apoptosis through blockade of the BCR-PLC*γ*2-NF-*κ*B signaling axis, and indirect suppression of hematopoietic progenitor cells via inhibition of bone marrow stromal cytokines and TEC family kinases, ultimately leading to neutropenia and thrombocytopenia (56).

Pirtobrutinib showed a markedly elevated reporting odds ratio (ROR = 54.28) for increased white blood cell count, which represents a benign pharmacodynamic effect rather than a toxic endpoint. The BRUIN trial reported elevated white blood cell counts in 46% of CLL/SLL (57). Our finding of an exceptionally high ROR (54.28) for this event translates this frequent pharmacodynamic effect into a disproportionality signal, confirming its prominence relative to the background reporting rate of all AEs in FAERS. This underscores that it is a class-effect for BTK inhibitors but is most pronounced with pirtobrutinib. According to the National Comprehensive Cancer Network (NCCN) guidelines (58), pirtobrutinib, a non-covalent BTK inhibitor, is recommended for patients with BTK C481S mutations, though real-world safety data remain limited. This study indicates that despite prominent hematological signals, pirtobrutinib's risks remain manageable. To address the existing gaps in its safety evidence, larger and more rigorously designed post-marketing studies are warranted.

Based on these findings, the following monitoring strategy of BTK inhibitor is recommended: (1) Obtain complete blood count and coagulation profiles at baseline prior to treatment initiation (59); (2) Monitor blood counts every 2–4 weeks during therapy, if grade ≥3 neutropenia (<1.0 × 10^9^/L) or thrombocytopenia (<50 × 10^9^/L) is documented on three or more occasions, treatment should be discontinued and supportive care with granulocyte-colony stimulating factor (G-CSF) or thrombopoietin-receptor agonists (TPO-RA) initiated (60); (3) For patients with a history of myelosuppression, acalabrutinib may be preferred given its relatively favorable hematological safety profile, as current guidelines do not specify myelotoxicity priorities among BTK inhibitors.

#### 4.2.3 Cardiac disorders

Cardiac disorders represented the safety domain with the most pronounced differences in disproportionality reporting patterns among BTK inhibitors. Ibrutinib demonstrated the strongest cardiovascular-related disproportionality signals, particularly for atrial fibrillation, as reflected by elevated signal strength and a temporally scattered distribution pattern. The observed distribution pattern of atrial fibrillation reports suggests that these events were reported across different treatment-duration intervals rather than being restricted to an early reporting period. Additionally, cardiac failure associated with ibrutinib showed a statistically significant association with reported fatal outcomes, highlighting the importance of further evaluating this signal in clinical studies. The mechanisms linking ibrutinib and atrial fibrillation remain incompletely understood, though evidence supports both target and off-target pathways. Inhibition of C-terminal Src kinase (CSK) may play a pivotal role in atrial fibrillation development. CSK is enriched in atrial myocardium compared to ventricular tissue, and CSK deficiency has been shown to promote atrial fibrillation, cardiac fibrosis, and inflammation (e.g., IL-6-mediated inflammation) (61).

In contrast, acalabrutinib and zanubrutinib exhibited attenuated cardiac signals, with lower signal strengths for atrial fibrillation than ibrutinib. Pirtobrutinib showed no atrial fibrillation signal among its top 30 PTs, with only four reported cases of cardiac failure. Although limited sample size warrants cautious interpretation, the overall data indicate a favorable cardiotoxicity profile for pirtobrutinib. These findings align with and extend the evidence from head-to-head randomized trials [e.g., ELEVATE-RR (10), ALPINE (11)] by demonstrating the persistent and strong association of ibrutinib with atrial fibrillation in a much larger, heterogeneous patient population. The high ROR and its temporal distribution pattern provide real-world validation of the trial results and emphasize the need for sustained cardiovascular monitoring as recommended by guidelines. Among patients switching to pirtobrutinib due to intolerance to other BTK inhibitors, atrial fibrillation recurrence rates remained below 1% (62). Mechanistically, these inhibitors achieve high selectivity for BTK while minimizing off-target inhibition of kinases such as ITK, TEC, and BMX, thereby preserving PI3K-Akt-eNOS-Ca²⁺ signaling homeostasis in myocardial tissue (63). This selective targeting reduces the risk of atrial fibrillation and life-threatening ventricular arrhythmias, resulting in significantly lower cardiotoxicity compared to ibrutinib. These findings may support enhanced cardiovascular monitoring considerations for patients receiving BTK inhibitors, particularly those with relevant clinical risk factors. Prior to treatment initiation, electrocardiogram (ECG) and echocardiographic assessment is recommended. During therapy, cardiac electrical activity and blood pressure should be monitored every 4–8 weeks (64). In cases of grade ≥3 atrial fibrillation or ventricular arrhythmia, prompt treatment discontinuation and multidisciplinary management are advised (65), which may substantially mitigate BTK inhibitor-related cardiotoxicity.

#### 4.2.4 Infections and infestations

Infections and infestations represent a critical safety concern in BTK inhibitor therapy, warranting focused clinical attention. While all inhibitors showed significant signals at the SOC level, distinct agent-specific disproportionality signal patterns were observed. Ibrutinib exhibited a late-concentrated reporting pattern for infection-related adverse events, exemplified by pneumonia incidence transitioning from a scattered early distribution (16.5% in 0–30 days) to a concentrated late-phase pattern (40.1% after 360 days), reflecting a greater proportion of infection-related reports among cases with longer reported treatment durations. This pattern may be mechanistically linked to ibrutinib's immunosuppressive effects: long-term use induces multi-axis suppression via BCR-macrophage-TLR/FC*γ*R-Th1 pathways (66), causing persistent dysfunction of neutrophils, macrophages, and T-cells that cumulatively elevates infection risk. Additionally, ibrutinib demonstrated signals for urinary tract infections, which were generally mild and rarely required treatment discontinuation, addressing a gap in current guidelines regarding long-term risk monitoring.

Acalabrutinib demonstrated the weakest infection-related reporting signal among the four agents, consistent with the ELEVATE-RR trial (10) showing significantly lower incidence of grade ≥ 3 infections compared to ibrutinib. Zanubrutinib showed the strongest overall SOC-level for infections, though its pneumonia-specific risk was comparable to acalabrutinib. This apparent discrepancy reflects zanubrutinib's broader infection spectrum, as clarified by the ALPINE trial (11) where higher rates of COVID-19 and upper respiratory tract infections were observed compared to ibrutinib. Mechanistically, both acalabrutinib and zanubrutinib achieve high BTK selectivity while preserving off-target kinases like ITK and TEC, maintaining macrophage TLR/Fc*γ*R signaling and T-cell IFN-*γ* responses (67), which underlies their relatively lower pneumonia incidence.

Although pirtobrutinib was associated with a relatively low absolute number of pneumonia reports, its robust signal strength—evidenced by a ROR = 4.53 and EBGM = 4.44—underscores that its infection risk should not be underestimated. This observation aligns with Phase I/II trial data, in which infections were the most common AE, occurring in 71.0% of patients. Furthermore, as a highly selective non-covalent BTK inhibitor, pirtobrutinib's infection risk profile may be influenced by additional factors, particularly its predominant use in patients with relapsed/refractory B-cell malignancies who have typically undergone multiple prior therapies, leading to cumulative immune impairment and thus a more vulnerable baseline immune status.

While European Society for Medical Oncology (ESMO) Guidelines (2024) (68) recommend infection monitoring every 3–6 months for all BTK inhibitors, agent-specific risk profiles suggest intensified monitoring for ibrutinib and zanubrutinib patients. For high-risk patients initiating acalabrutinib or zanubrutinib, comprehensive management should include: pre-treatment assessment of vaccination status, serum IgG levels, and baseline chest x-ray imaging; regular monitoring of blood counts and infection markers every 4–8 weeks during therapy; prophylactic intravenous immunoglobulin and trimethoprim-sulfamethoxazole for patients with serum IgG < 5 g/L or recurrent pneumonia; and immediate therapy suspension of with targeted anti-infectives, granulocyte colony-stimulating factor (G-CSF), and immunoglobulin support for grade ≥3 infections per established guidelines (69). The differential infection risks observed across the BTK inhibitors have direct implications for implementing ESMO guideline recommendations (68). The guideline's general advice for infection monitoring can be optimized based on our findings: for patients on ibrutinib or zanubrutinib, who showed higher or broader infection signals, a more vigilant approach aligned with our proposed strategy may be warranted. In contrast, for acalabrutinib patients, standard monitoring may suffice, reflecting its more favorable infection profile in both trials (10) and this real-world analysis.

#### 4.2.5 Safety characteristics in additional SOCs and AE-death associations

Other SOCs also revealed notable safety signals warranting supplementary analysis to enhance monitoring frameworks. Zanubrutinib showed the highest ROR for haemorrhage subcutaneous among the four inhibitors. Concurrent use with anticoagulants or antiplatelet agents necessitates vigilant monitoring, underscoring the need for pre-treatment bleeding risk assessment (70). Ibrutinib showed elevated ROR for contusion, indicating a generalized bleeding tendency beyond subcutaneous sites; patients should be advised to avoid trauma during therapy. BTK, a key component of the BCR signaling pathway, is also involved in platelet signaling pathways. When BTK inhibitors exert their effects, they suppress not only BTK in B cells but also platelet function. First-generation BTK inhibitors such as ibrutinib, due to their relatively low selectivity, also inhibit TEC kinase. Both BTK and TEC play important roles in platelet activation signals mediated by the collagen receptor Glycoprotein VI (GPVI) and Fc receptors. Dual inhibition impedes platelet activation and aggregation, thereby increasing the risk of bleeding.

Diarrhea risk was higher with first-generation BTK inhibitors like ibrutinib compared to second- and third-generation agents. Although the association between diarrhea and death was weak for ibrutinib, severe cases may lead to complications such as dehydration and electrolyte imbalance, warranting rehydration and antidiarrheal therapy for grade ≥2 events. Other real-world studies (71) report that 11% of patients require hospitalization due to diarrhea, with grade ≥2 events independently associated with elevated 30-day mortality. Acalabrutinib demonstrated a relatively high ROR for headache, which carried the highest clinical priority among the four drugs and showed a mortality association. While typically mild, serious underlying causes such as intracranial haemorrhage or infection must be excluded, particularly in hypertensive patients. Given the limited report volume for pirtobrutinib, a notable disproportionality signal for malignant neoplasm progression was observed in this analysis, reflecting its use in relapsed/refractory patients with high baseline tumor burden. Regular imaging is essential to differentiate drug-associated progression from natural disease course; current guidelines recommend CT or MRI assessments every 8 weeks for such differentiation (72).

Regarding the potential follow-up bias due to differential marketing durations, we applied a standardized two-year observation window from the first quarter of 2023 to the first quarter of 2025 to mitigate the impact of varying surveillance periods and we acknowledge that residual bias may still persist. This is primarily because the intensity of adverse event reporting often differs between established and newly approved agents. We further considered the applicability of alternative data-selection strategies to address this issue. Incidence density sampling is theoretically superior as it analyzes the incidence density of events per person-time at risk rather than simple reporting ratios, yet the FAERS database lacks reliable denominator data on the number of patients exposed to each drug and renders such calculation infeasible. Reference date matching represents another more rigorous approach that aligns the post-approval timeline of newer agents with the early-phase data of older counterparts, but this method may introduce historical bias given that background medical practices including supportive care and concomitant medications have evolved greatly over the years. Under the inherent constraints of spontaneous reporting systems, the adoption of a two-year observation window combined with multi-algorithm cross-validation remains the most pragmatic solution to balance sufficient data volume and temporal comparability.

The subgroup analysis revealed that the core safety signals for ibrutinib, acalabrutinib, and zanubrutinib remained consistent across both serious event and first-line treatment patient populations. This significantly enhances the robustness of the identified signals, indicating that they reflect the drugs' inherent safety profiles and can manifest early in the course of treatment. The presence of reporting bias and unmeasured patient characteristic differences between subgroups means that these results primarily provide hypotheses requiring priority validation, rather than constituting conclusive evidence of comparative risk.

This study enhanced the credibility of its core findings by introducing a negative control analysis. The absence of significant signals for the negative control outcomes provides important methodological support for the specificity of the primary positive signals, indicating that these signals are more likely to reflect genuine drug-event associations rather than pervasive reporting bias.

The association between reported AEs and fatal outcomes provides additional information regarding adverse event patterns observed among fatal reports in FAERS. Ibrutinib exhibited strong associations between fatal outcomes and several high-risk PTs, including disease progression, respiratory failure, and sepsis. Additionally, cardiovascular PTs such as cardiac failure and myocardial infarction also demonstrated significant mortality associations, further highlighting cardiovascular-related fatal outcome signals associated with ibrutinib reports. In contrast, acalabrutinib showed fewer PTs with significant mortality associations, and the overall association strength was comparatively weak, with only headache, diarrhea, and dyspnea demonstrating notable links to death. Zanubrutinib demonstrated no significant association between its reported PTs and fatal outcomes, and combined with a death report ratio of 7.7%, these findings indicate no statistically significant disproportionality signal for reported fatal outcomes in this dataset. Pirtobrutinib exhibited a high ROR for the death PT, though this was based on only 50 reports; the limited sample size compromises signal robustness, and no PTs with clearly elevated fatal risk were identified, necessitating further validation through larger studies.

To facilitate interpretation of the identified reporting signals, the core safety findings and monitoring considerations for the four BTK inhibitors are summarized in Table 5. In summary, this study refines the reporting safety profiles of BTK inhibitors by integrating association strength, temporal characteristics, and drug positioning. The observed signal differentials provide clues for formulating agent-specific monitoring priorities in clinical practice.

**Table 5:** Key characteristics and clinical management strategies of four BTK inhibitors.

BTK Inhibitor	Key Characteristics	Clinical Monitoring Considerations
Ibrutinib	Highest cardiotoxicity risk Infection risk accumulates with prolonged exposure Cardiac events show significant association with fatal outcomes	Baseline: ECG, echocardiogram, complete blood count, coagulation profile, IgG level Monitoring: Cardiac function; infection markers Intervention: Discontinue for grade ≥3 atrial fibrillation/ventricular arrhythmia; initiate G-CSF for grade ≥3 neutropenia; provide prophylactic antibiotics for high-risk patients Selection: Reduce use in patients with cardiovascular disease
Acalabrutinib	Attenuated off-target toxicity Most favorable overall safety profile Headache is the most common AE with potential mortality association	Baseline: Complete blood count, coagulation profile Monitoring: infection Intervention: Rule out intracranial hemorrhage/infection for severe headache; symptomatic treatment for mild events Selection: Elderly patients, those with comorbidities, or history of myelosuppression
Zanubrutinib	Strongest myelosuppressive signal among four agents Unique subcutaneous bleeding risk Broader infection spectrum	4. Baseline: complete blood count, coagulation profile, bleeding risk 5. Monitoring: intensified infection 6. Intervention: Discontinue for persistent grade ≥3 cytopenia 7. Selection: Use with caution in patients with bleeding diathesis or baseline cytopenia
Pirtobrutinib	Highest ROR for increased white blood cell count (benign pharmacodynamic effect) Limited post-marketing data Predominantly used in heavily pretreated relapsed/refractory patients	Baseline: Complete blood count, imaging assessment of tumor burden Monitoring: Regular complete blood count and imaging Intervention: Differentiate pharmacodynamic leukocytosis from disease progression Selection: Reserved for patients with BTK C481S mutations or intolerance to covalent BTK inhibitors

The table is intended only as a reference for clinical safety monitoring and should not be used as a basis for treatment selection.

### 4.3 Limitations of this study

This study, utilizing real-world data from the FAERS database, provides a descriptive pharmacovigilance analysis of AE reporting signals among four BTK inhibitors. Nevertheless, several limitations warrant consideration: (1) Reporting and Missing Data Bias: Significant disparities were observed in the volume of AE reports among the four BTK inhibitors. Although sensitivity analyses were conducted using a standardized reporting time window, the potential influence of differences in reporting intensity among agents on signal detection cannot be completely excluded. In clinical practice, healthcare professionals may be more likely to report severe or clinically significant events, resulting in potential overrepresentation of serious cases and underrepresentation of milder events in FAERS. Additionally, zanubrutinib exhibited notably higher missing data rates for gender and age variables compared to the other three inhibitors. Although Little's MCAR test did not provide sufficient statistical evidence against the MCAR assumption, the extremely high proportion of missing demographic information for zanubrutinib substantially limits the ability to verify the missingness mechanism. Multiple imputation was therefore performed only as an exploratory approach, and demographic-related findings should be interpreted cautiously. Furthermore, recently approved drugs may be subject to stimulated reporting due to increased clinical attention and enhanced post-marketing surveillance, which may influence observed disproportionality signals. (2) Insufficient Control of Confounding Factors: The FAERS database does not systematically capture detailed information on concomitant medications or underlying comorbidities. These factors may influence observed adverse event reporting patterns and associations with fatal outcomes, potentially affecting interpretation of disproportionality signals. (3) Lack of Objective Laboratory Data Verification: The FAERS database lacks objective laboratory or auxiliary examination results, including complete blood counts, liver/kidney function tests, electrocardiograms, or imaging studies. Diagnosis and severity classification of AEs rely largely on reporter-provided information without standardized laboratory or clinical validation, which may introduce outcome misclassification and influence estimated disproportionality signal strength. Therefore, this study can only identify disproportionality reporting signals, and cannot estimate actual incidence rates or establish direct comparative safety risks between different BTK inhibitors. All cross-drug comparisons in this study represent differences in disproportionality reporting signal patterns and should not be interpreted as confirmed differences in clinical safety risk.

### 4.4 Future research directions

Future studies should pursue more comprehensive comparative safety assessments through the integration of multiple real-world data sources. Integrating databases such as Sentinel, Medicare, NHIRD, and other healthcare insurance systems can provide complete, closed-loop data that encompass prescription doses, exposure person-time, and cause-of-death certifications. Such integration would enable estimation of real-world incidence rates, standardized mortality ratios, and comparative outcome measures associated with BTK inhibitor therapy. The treatment landscape for BTK inhibitors has evolved from monotherapy to diverse combination regimens, including BTK inhibitors combined with immune checkpoint inhibitors, chemotherapy agents, or other targeted therapies in dual-targeted approaches. However, safety data for these combination therapies remain limited and warrant systematic evaluation in future clinical investigations. BTK inhibitors were approved in 2025 for the treatment of autoimmune diseases, including Primary Immune Thrombocytopenia and Chronic Spontaneous Urticaria. However, more clinical data are still needed to better characterize their long-term effectiveness and safety patterns in these immune-mediated conditions. Therefore, forthcoming research should prioritize addressing existing evidence gaps and aligning with unmet clinical needs. By integrating multi-source data and conducting rigorous safety evaluations of combination therapies, the evidence base regarding BTK inhibitor safety and effectiveness can be substantially strengthened. This approach will facilitate more accurate comparative safety assessment and long-term pharmacovigilance monitoring.

## 5 Conclusions

This study presents a descriptive, hypothesis-generating pharmacovigilance analysis of four BTK inhibitors using the FAERS database, revealing distinct differences in disproportionality reporting signal patterns among different generations of BTK inhibitors. While all agents shared common disproportionality reporting signal domains at the SOC level—specifically Blood and Lymphatic System Disorders, Cardiac Disorders, and Infections and Infestations—distinct PT-level disproportionality reporting patterns were observed among individual BTK inhibitors. The first-generation inhibitor ibrutinib demonstrated a strong cardiovascular-related disproportionality reporting signal, particularly for atrial fibrillation. Second-generation inhibitors acalabrutinib and zanubrutinib showed weaker atrial fibrillation-related disproportionality signals compared with ibrutinib in this dataset. They also demonstrated distinct reporting patterns, with acalabrutinib showing a notable headache-related signal and zanubrutinib showing additional signals involving myelosuppression-related and subcutaneous haemorrhage-related events. The third-generation inhibitor pirtobrutinib displayed a prominent disproportionality signal for increased white blood cell count, which has been described as a potential pharmacodynamic effect in previous studies. Regarding reported fatal outcomes, ibrutinib-associated reports showed stronger statistical associations between multiple PTs and fatal outcomes, whereas fewer significant associations were observed for acalabrutinib. Additionally, descriptive temporal analysis demonstrated distinct distributions of reported adverse event onset intervals: decreased white blood cell count and haemorrhage reports associated with ibrutinib were relatively concentrated within earlier reported intervals, whereas pneumonia-related reports represented a larger proportion among cases with longer reported treatment durations. Atrial fibrillation reports were distributed across multiple reported treatment intervals. Pirtobrutinib demonstrated a relatively early-concentrated temporal reporting pattern, with a higher proportion of reported adverse events within the 0–30 day interval followed by fewer reports in subsequent intervals. These findings provide potential signals for post-marketing safety surveillance and generate hypotheses for further clinical investigation. However, the identified disproportionality patterns should not be interpreted as evidence of confirmed differences in clinical safety risk or used alone to guide treatment selection. Future research should further evaluate these observations through integration of multi-source real-world data, comparative effectiveness studies, and systematic assessment of combination therapy safety.

## Data Availability

The original contributions presented in the study are included in the article/Supplementary Material, further inquiries can be directed to the corresponding authors.
