# Supplementary material for: Generational safety profiles of BTK inhibitors: adverse reaction signals and real-world evidence from the FAERS database
Source: Front Med (Lausanne). 2026 Sep 4;13:1832040. doi: 10.3389/fmed.2026.1832040 (PMC13585522; doi:10.3389/fmed.2026.1832040)
Supplement: Supplementary file 1 [file Table1.docx]

Supplementary Material

Supplementary Table 1. 2 × 2 Contingency Table for Drug-Adverse Event Association Analysis

| **Group** | **Adverse Event Occurred (Positive)** | **Adverse Event Absent (Negative)** | **Total** |
| --- | --- | --- | --- |
| BTK Inhibitor Group | a | b | a + b |
| Non-BTK Inhibitor Group | c | d | c+ d |
| Total | a + c | b+d | N |
| BTK: Bruton’s tyrosine kinase | | | |

Supplementary Table 2. Safety Signal Detection Methods and Thresholds

| **Method** | **Core Indicator** | **Calculation Formula** | **Threshold Criteria** | **Applicability** |
| --- | --- | --- | --- | --- |
| ROR | ROR  (95% CI) | ROR = $\frac{\frac{\text{a}}{\text{c}}}{\frac{\text{b}}{\text{d}}}$  $\text{SE(lnROR)}$ = $\sqrt{\text{(}\frac{\text{1}}{\text{a}}\text{) + (}\frac{\text{1}}{\text{b}}\text{) + (}\frac{\text{1}}{\text{c}}\text{) + (}\frac{\text{1}}{\text{d}}\text{)}}$  95% CI = $\text{e}^{\text{lnROR }\text{± 1.96 × SE(lnROR)]}}$ | a ≥ 3 and 95% CI lower bound > 1 | Large-sample data, high sensitivity |
| PRR & MHRA Comprehensive Criteria | PRR (χ²) | PRR = $\frac{\frac{\text{a}}{\text{a}\text{ + }\text{b}}}{\frac{\text{c}}{\text{c}\text{ + }\text{d}}}$  SE(lnPRR) = $\sqrt{\text{(}\frac{\text{1}}{\text{a}}\text{) - (}\frac{\text{1}}{\text{a}\text{ + }\text{b}}\text{) + (}\frac{\text{1}}{\text{c}}\text{) - (}\frac{\text{1}}{\text{c}\text{-}\text{d}}\text{)}}$  95% CI = $\text{e}^{\text{lnPRR }\text{± 1.96 × SE(lnPRR)]}}$  χ² = $\frac{\begin{aligned} \text{(}\text{ad}\text{ - }\text{bc}\text{)}^{\text{2}}\text{(}\text{a}\text{ + }\text{b}\text{ + }\text{c}\text{ } \\ \text{+ }\text{d}\text{)} \end{aligned}}{\text{(}\text{a}\text{ + }\text{b}\text{)(}\text{a}\text{ + }\text{c}\text{)(}\text{c}\text{ + }\text{d}\text{)(}\text{b}\text{ + }\text{d}\text{)}}$ | PRR ≥ 2, χ² ≥ 4,  and a ≥ 3 | Medium / high-frequency AEs |
| MGPS (EBGM) | EBGM (EBGM_05_) | EBGM = $\frac{\text{a}\text{(}\text{a}\text{ + }\text{b}\text{ + }\text{c}\text{ + }\text{d}\text{)}}{\text{(}\text{a}\text{ + }\text{c}\text{)(}\text{a}\text{ + }\text{b}\text{)}}$  $\text{SE(lnEBGM)}$ = $\sqrt{\text{(}\frac{\text{1}}{\text{a}}\text{) + (}\frac{\text{1}}{\text{b}}\text{) + (}\frac{\text{1}}{\text{c}}\text{) + (}\frac{\text{1}}{\text{d}}\text{)}}$  EBGM_05_ = 95% CI = $\text{e}^{\text{lnEBGM }\text{± 1.96 × SE(lnEBGM)]}}$ | EBGM_05_ ≥ 2 and  n ≥ 3 | Rare AEs (small-sample stability) |
| BCPNN (IC) | IC (IC_025_) | α_i_ = β_j_ = 1, α = β = 2, γ_ij_ = 1  N ＝ a + b + c + d $\text{IC}=\log_{2}\frac{a\text{(}a+b+c+d\text{)}}{\text{(}a+b\text{)(}a+c\text{)}}$ $\text{SD}=\sqrt{\text{V(IC)}}$           IC_025_=E(IC)-2SD | IC_025_ > 0 | Heterogeneous / multi-center data |

ROR: Reporting Odds Ratio; PRR: Proportional Reporting Ratio; MHRA: Medicines and Healthcare products Regulatory Agency; MGPS: Multi-item Gamma Poisson Shrinker; EBGM: Empirical Bayes Geometric Mean; CI: Confidence Interval; χ²: Chi-Square Statistic; IC: Information Component; α, αi, β, βi, γij: are all hyperparameters; SD: Standard Deviation.

Supplementary Table 3. Missing Rates of Core Variables for the Four BTK Inhibitors

| **Variable** | **Ibrutinib**  **(n = 67,095)** | **Acalabrutinib**  **(n = 7,886)** | **Zanubrutinib**  **(n = 1,810)** | **Pirtobrutinib**  **(n = 223)** |
| --- | --- | --- | --- | --- |
| **Sex** | 6.3% | 30.7% | **97.2%** | 23.3% |
| **Age** | 53.9% | 66.0% | **91.9%** | 46.6% |
| **Reporting Country** | 6.7% | ~0.0% | 0.1% | 0.0% |
| **Outcomes** | 0.0% | 0.0% | 0.0% | 0.0% |
| Serious cases | 0.0% | 0.0% | 0.0% | 0.0% |

Outcomes and Serious Cases have no missing data by FAERS reporting rules (mandatory fields). For acalabrutinib, the missing rate for "Reporting Country" is calculated as 3 / 7,886 × 100% = 0.038% (rounded to 0.0% per one decimal place convention).

| Supplementary Table 4. Baseline Characteristic Distributions of Patients with the Four BTK Inhibitors After Multiple Imputation | | | | | | | | |
| --- | --- | --- | --- | --- | --- | --- | --- | --- |
| **Characteristic** | ****Ibrutinib**** | | **Acalabrutinib** | | **Zanubrutinib** | | **Pirtobrutinib** | |
|  | **n** | **%** | **n** | **%** | **n** | **%** | **n** | **%** |
| Sex |  |  |  |  |  |  |  |  |
| Male | 41465 | 61.8 | 4976 | 63.1 | 1323 | 73.1 | 170 | 76.2 |
| Female | 25630 | 38.2 | 2910 | 36.9 | 487 | 26.9 | 53 | 23.8 |
| Age (years) |  |  |  |  |  |  |  |  |
| ＜18 | 100 | 0.15 | 0 | 0 | 0 | 0 | 0 | 0 |
| 18~64 | 11272 | 16.8 | 1388 | 17.6 | 439 | 24.2 | 51 | 23.1 |
| 65~85 | 49583 | 73.9 | 4890 | 62.0 | 1208 | 66.7 | 145 | 64.6 |
| >85 | 6140 | 9.15 | 1608 | 20.4 | 163 | 9.0 | 27 | 12.3 |

Supplementary Table 5-A. System Organ Class (SOC)-Level Safety Signals for Ibrutinib

| **Ibrutinib SOC** | **Case Reports** | **ROR (95% CI)** | **PRR (χ^2^)** | **EBGM (EBGM_05_)** | **IC (IC_025_)** |
| --- | --- | --- | --- | --- | --- |
| General disorders and administration site conditions | 31247 | 0.82 (0.81-0.83) | 0.84 (1103.16) | 0.84 (0.83) | -0.25 (-0.26) |
| Injury, poisoning and procedural complications | 20752 | 0.92 (0.91-0.93) | 0.93 (131.90) | 0.93 (0.91) | -0.11 (-0.13) |
| Gastrointestinal disorders | 17732 | 1.03 (1.01-1.04) | 1.02 (10.47) | 1.02 (1.01) | 0.03 (0.01) |
| Infections and infestations | 16912 | 1.55 (1.53-1.58) | 1.51 (3043.56) | 1.51 (1.48) | 0.59 (0.57) |
| Investigations | 16877 | 1.45 (1.43-1.47) | 1.41 (2161.03) | 1.41 (1.39) | 0.5 (0.47) |
| Nervous system disorders | 12400 | 0.74 (0.72-0.75) | 0.75 (1094.05) | 0.75 (0.74) | -0.41 (-0.44) |
| Skin and subcutaneous tissue disorders | 11665 | 1 (0.99-1.02) | 1 (0.22) | 1 (0.99) | 0.01 (-0.02) |
| Musculoskeletal and connective tissue disorders | 11434 | 1.07 (1.05-1.09) | 1.06 (46.51) | 1.06 (1.04) | 0.09 (0.06) |
| Respiratory, thoracic and mediastinal disorders | 10796 | 1.13 (1.1-1.15) | 1.12 (142.54) | 1.12 (1.1) | 0.16 (0.13) |
| Cardiac disorders | 9693 | 2.17 (2.13-2.22) | 2.12 (5794.69) | 2.11 (2.06) | 1.08 (1.05) |
| Surgical and medical procedures | 8551 | 2.98 (2.91-3.04) | 2.9 (10617.01) | 2.87 (2.81) | 1.52 (1.49) |
| Blood and lymphatic system disorders | 6779 | 2.04 (1.99-2.09) | 2.01 (3443.53) | 2 (1.95) | 1 (0.96) |
| Vascular disorders | 5522 | 1.35 (1.31-1.39) | 1.34 (485.76) | 1.34 (1.3) | 0.42 (0.38) |
| Neoplasms benign, malignant and unspecified (incl cysts and polyps) | 5307 | 0.9 (0.88-0.93) | 0.9 (56.72) | 0.9 (0.88) | -0.15 (-0.19) |
| Metabolism and nutrition disorders | 4241 | 0.99 (0.96-1.02) | 0.99 (0.22) | 0.99 (0.96) | -0.01 (-0.05) |
| Psychiatric disorders | 12400 | 0.36 (0.35-0.37) | 0.37 (4636.38) | 0.37 (0.36) | -1.43 (-1.47) |
| Eye disorders | 4111 | 1 (0.97-1.03) | 1 (0.06) | 1 (0.97) | -0.01 (-0.05) |
| Renal and urinary disorders | 3868 | 0.99 (0.96-1.02) | 0.99 (0.22) | 0.99 (0.96) | -0.01 (-0.06) |
| Immune system disorders | 1387 | 0.57 (0.54-0.6) | 0.57 (459.81) | 0.57 (0.54) | -0.81 (-0.89) |
| Ear and labyrinth disorders | 1173 | 1.31 (1.24-1.39) | 1.31 (86.05) | 1.31 (1.24) | 0.39 (0.3) |
| Hepatobiliary disorders | 1118 | 0.65 (0.61-0.68) | 0.65 (215.99) | 0.65 (0.61) | -0.63 (-0.71) |
| Reproductive system and breast disorders | 808 | 0.51 (0.47-0.54) | 0.51 (386.98) | 0.51 (0.48) | -0.97 (-1.07) |
| Social circumstances | 351 | 0.39 (0.35-0.44) | 0.39 (327.4) | 0.4 (0.36) | -1.34 (-1.49) |
| Endocrine disorders | 286 | 0.54 (0.48-0.6) | 0.54 (114.63) | 0.54 (0.48) | -0.9 (-1.06) |
| Product issues | 258 | 0.07 (0.06-0.08) | 0.07 (3346.99) | 0.07 (0.06) | -3.87 (-4.04) |
| Congenital, familial and genetic disorders | 107 | 0.19 (0.16-0.23) | 0.19 (357.38) | 0.2 (0.16) | -2.36 (-2.62) |
| Pregnancy, puerperium and perinatal conditions | 5 | 0.01 (0-0.01) | 0.01 (801.82) | 0.01 (0) | -7.33 (-8.25) |

Supplementary Table 5-B. SOC-Level Safety Signals for Acalabrutinib

| **Acalabrutinib SOC** | **Case Reports** | **ROR (95% CI)** | | **PRR (χ^2^)** | | **EBGM (EBGM_05_)** | | **IC (IC_025_)** | |  |
| --- | --- | --- | --- | --- | --- | --- | --- | --- | --- | --- |
| General disorders and administration site conditions | 4023 | | 1.28 (1.24-1.33) | | 1.22 (199.57) | | 1.22 (1.18) | | 0.29 (0.24) | |
| Investigations | 1641 | | 1.58 (1.5-1.66) | | 1.53 (317.37) | | 1.53 (1.45) | | 0.61 (0.54) | |
| Nervous system disorders | 1556 | | 1.12 (1.06-1.18) | | 1.11 (17.48) | | 1.11 (1.05) | | 0.15 (0.07) | |
| Injury, poisoning and procedural complications | 1535 | | 0.66 (0.62-0.69) | | 0.69 (250.68) | | 0.69 (0.65) | | -0.54 (-0.62) | |
| Gastrointestinal disorders | 1363 | | 0.9 (0.85-0.95) | | 0.91 (14.17) | | 0.91 (0.86) | | -0.14 (-0.22) | |
| Infections and infestations | 1063 | | 1.02 (0.96-1.09) | | 1.02 (0.56) | | 1.02 (0.96) | | 0.03 (-0.06) | |
| Musculoskeletal and connective tissue disorders | 923 | | 0.99 (0.92-1.05) | | 0.99 (0.14) | | 0.99 (0.92) | | -0.02 (-0.11) | |
| Respiratory, thoracic and mediastinal disorders | 884 | | 1.04 (0.97-1.11) | | 1.03 (1.07) | | 1.03 (0.97) | | 0.05 (-0.05) | |
| Cardiac disorders | 850 | | 2.31 (2.16-2.48) | | 2.25 (604.74) | | 2.25 (2.1) | | 1.17 (1.07) | |
| Skin and subcutaneous tissue disorders | 758 | | 0.67 (0.63-0.72) | | 0.69 (115.75) | | 0.69 (0.64) | | -0.54 (-0.65) | |
| Blood and lymphatic system disorders | 750 | | 2.44 (2.27-2.63) | | 2.38 (611.2) | | 2.38 (2.21) | | 1.25 (1.14) | |
| Neoplasms benign, malignant and unspecified (incl cysts and polyps) | 749 | | 1.24 (1.16-1.34) | | 1.23 (34.41) | | 1.23 (1.15) | | 0.3 (0.2) | |
| Vascular disorders | 531 | | 1.53 (1.4-1.67) | | 1.51 (94.05) | | 1.51 (1.39) | | 0.6 (0.47) | |
| Metabolism and nutrition disorders | 484 | | 1.32 (1.21-1.44) | | 1.31 (36.35) | | 1.31 (1.2) | | 0.39 (0.26) | |
| Psychiatric disorders | 417 | | 0.41 (0.37-0.45) | | 0.42 (347.44) | | 0.42 (0.38) | | -1.24 (-1.38) | |
| Renal and urinary disorders | 351 | | 0.94 (0.85-1.04) | | 0.94 (1.35) | | 0.94 (0.85) | | -0.09 (-0.24) | |
| Eye disorders | 251 | | 0.68 (0.6-0.77) | | 0.69 (36.91) | | 0.69 (0.61) | | -0.54 (-0.73) | |
| Immune system disorders | 152 | | 0.66 (0.56-0.78) | | 0.66 (26.18) | | 0.66 (0.57) | | -0.59 (-0.82) | |
| Hepatobiliary disorders | 141 | | 0.89 (0.75-1.05) | | 0.89 (1.92) | | 0.89 (0.75) | | -0.17 (-0.41) | |
| Ear and labyrinth disorders | 138 | | 1.76 (1.49-2.08) | | 1.75 (44.96) | | 1.75 (1.48) | | 0.81 (0.56) | |
| Reproductive system and breast disorders | 61 | | 0.5 (0.39-0.65) | | 0.51 (29.55) | | 0.51 (0.39) | | -0.98 (-1.34) | |
| Surgical and medical procedures | 57 | | 0.21 (0.16-0.27) | | 0.21 (175.01) | | 0.21 (0.16) | | -2.27 (-2.63) | |
| Social circumstances | 36 | | 0.4 (0.29-0.56) | | 0.41 (31.41) | | 0.41 (0.29) | | -1.3 (-1.75) | |
| Endocrine disorders | 23 | | 0.46 (0.31-0.7) | | 0.47 (14.18) | | 0.47 (0.31) | | -1.1 (-1.66) | |
| Product issues | 23 | | 0.06 (0.04-0.1) | | 0.07 (312.2) | | 0.07 (0.04) | | -3.93 (-4.46) | |
| Congenital, familial and genetic disorders | 21 | | 0.45 (0.29-0.69) | | 0.45 (14.33) | | 0.45 (0.29) | | -1.16 (-1.74) | |
| Pregnancy, puerperium and perinatal conditions | 2 | | 0.03 (0.01-0.12) | | 0.03 (65.48) | | 0.03 (0.01) | | -5.11 (-6.21) | |

Supplementary Table 5-C. SOC-Level Safety Signals for Zanubrutinib

| **Zanubrutinib SOC** | **Case Reports** | **ROR (95% CI)** | **PRR (χ^2^)** | **EBGM (EBGM_05_)** | **IC (IC_025_)** |
| --- | --- | --- | --- | --- | --- |
| Injury, poisoning and procedural complications | 748 | 1.06 (0.98-1.14) | 1.05 (1.92) | 1.05 (0.97) | 0.07 (-0.04) |
| General disorders and administration site conditions | 739 | 0.69 (0.64-0.75) | 0.73 (86.84) | 0.73 (0.68) | -0.45 (-0.56) |
| Investigations | 599 | 1.93 (1.77-2.1) | 1.83 (239.75) | 1.83 (1.68) | 0.87 (0.75) |
| Infections and infestations | 516 | 1.66 (1.51-1.81) | 1.6 (121.99) | 1.6 (1.46) | 0.68 (0.54) |
| Skin and subcutaneous tissue disorders | 454 | 1.49 (1.35-1.64) | 1.45 (66.85) | 1.45 (1.32) | 0.54 (0.39) |
| Gastrointestinal disorders | 396 | 0.88 (0.79-0.97) | 0.89 (6.14) | 0.89 (0.8) | -0.17 (-0.32) |
| Nervous system disorders | 308 | 0.73 (0.65-0.82) | 0.75 (28.81) | 0.75 (0.66) | -0.42 (-0.59) |
| Blood and lymphatic system disorders | 293 | 3.13 (2.78-3.52) | 3.02 (402.43) | 3.02 (2.68) | 1.59 (1.41) |
| Respiratory, thoracic and mediastinal disorders | 283 | 1.1 (0.98-1.24) | 1.1 (2.56) | 1.1 (0.97) | 0.13 (-0.04) |
| Musculoskeletal and connective tissue disorders | 250 | 0.87 (0.77-0.99) | 0.88 (4.62) | 0.88 (0.77) | -0.19 (-0.38) |
| Cardiac disorders | 178 | 1.63 (1.4-1.89) | 1.61 (41.94) | 1.61 (1.39) | 0.69 (0.46) |
| Vascular disorders | 132 | 1.27 (1.06-1.5) | 1.26 (7.16) | 1.26 (1.06) | 0.33 (0.08) |
| Surgical and medical procedures | 125 | 1.45 (1.22-1.73) | 1.44 (17.19) | 1.44 (1.21) | 0.53 (0.26) |
| Metabolism and nutrition disorders | 118 | 1.08 (0.9-1.29) | 1.07 (0.62) | 1.07 (0.9) | 0.1 (-0.16) |
| Neoplasms benign, malignant and unspecified (incl cysts and polyps) | 113 | 0.53 (0.44-0.63) | 0.54 (47.12) | 0.54 (0.44) | -0.9 (-1.17) |
| Psychiatric disorders | 101 | 0.32 (0.26-0.39) | 0.33 (141.22) | 0.33 (0.27) | -1.58 (-1.86) |
| Renal and urinary disorders | 101 | 0.97 (0.8-1.18) | 0.97 (0.09) | 0.97 (0.8) | -0.04 (-0.33) |
| Eye disorders | 69 | 0.61 (0.48-0.78) | 0.62 (16.78) | 0.62 (0.49) | -0.7 (-1.04) |
| Hepatobiliary disorders | 44 | 0.92 (0.68-1.24) | 0.92 (0.31) | 0.92 (0.68) | -0.12 (-0.55) |
| Ear and labyrinth disorders | 36 | 1.55 (1.12-2.15) | 1.54 (6.93) | 1.54 (1.11) | 0.63 (0.13) |
| Immune system disorders | 35 | 0.53 (0.38-0.74) | 0.53 (14.35) | 0.53 (0.38) | -0.9 (-1.37) |
| Product issues | 22 | 0.19 (0.13-0.3) | 0.2 (73.2) | 0.2 (0.13) | -2.34 (-2.89) |
| Reproductive system and breast disorders | 15 | 0.43 (0.26-0.72) | 0.43 (11.13) | 0.43 (0.26) | -1.2 (-1.87) |
| Social circumstances | 13 | 0.47 (0.27-0.82) | 0.47 (7.61) | 0.47 (0.28) | -1.08 (-1.79) |
| Congenital, familial and genetic disorders | 3 | 0.22 (0.07-0.69) | 0.22 (8.15) | 0.22 (0.07) | -2.17 (-3.3) |
| Endocrine disorders | 3 | 0.2 (0.06-0.61) | 0.2 (9.86) | 0.2 (0.06) | -2.34 (-3.47) |
| Pregnancy, puerperium and perinatal conditions | 0 | - | - | - | - |

Supplementary Table 5-D. SOC-Level Safety Signals for Pirtobrutinib

| **Pirtobrutinib SOC** | **Case Reports** | **ROR (95% CI)** | **PRR (χ^2^)** | **EBGM (EBGM_05_)** | **IC (IC_025_)** |
| --- | --- | --- | --- | --- | --- |
| General disorders and administration site conditions | 109 | 1.45 (1.17-1.8) | 1.34 (11.59) | 1.34 (1.08) | 0.43 (0.11) |
| Investigations | 65 | 2.66 (2.04-3.46) | 2.42 (57.73) | 2.42 (1.86) | 1.28 (0.86) |
| Infections and infestations | 44 | 1.6 (1.18-2.19) | 1.55 (9.04) | 1.55 (1.13) | 0.63 (0.16) |
| Blood and lymphatic system disorders | 35 | 4.62 (3.27-6.52) | 4.34 (91.58) | 4.34 (3.07) | 2.12 (1.49) |
| Neoplasms benign, malignant and unspecified (incl cysts and polyps) | 26 | 3.19 (2.15-4.74) | 3.07 (36.88) | 3.07 (2.06) | 1.62 (0.94) |
| Injury, poisoning and procedural complications | 26 | 0.37 (0.25-0.55) | 0.4 (26.47) | 0.4 (0.27) | -1.31 (-1.85) |
| Nervous system disorders | 21 | 0.62 (0.4-0.96) | 0.64 (4.67) | 0.64 (0.41) | -0.65 (-1.26) |
| Gastrointestinal disorders | 21 | 0.54 (0.35-0.84) | 0.56 (7.8) | 0.56 (0.36) | -0.83 (-1.43) |
| Skin and subcutaneous tissue disorders | 18 | 0.69 (0.43-1.11) | 0.7 (2.36) | 0.7 (0.44) | -0.5 (-1.16) |
| Psychiatric disorders | 16 | 0.72 (0.44-1.19) | 0.73 (1.64) | 0.73 (0.45) | -0.45 (-1.14) |
| Cardiac disorders | 16 | 1.88 (1.14-3.1) | 1.85 (6.41) | 1.85 (1.13) | 0.89 (0.1) |
| Musculoskeletal and connective tissue disorders | 15 | 0.62 (0.37-1.04) | 0.64 (3.28) | 0.64 (0.38) | -0.65 (-1.35) |
| Respiratory, thoracic and mediastinal disorders | 12 | 0.55 (0.31-0.98) | 0.56 (4.29) | 0.56 (0.32) | -0.83 (-1.6) |
| Surgical and medical procedures | 9 | 1.15 (0.6-2.23) | 1.15 (0.18) | 1.15 (0.59) | 0.2 (-0.75) |
| Metabolism and nutrition disorders | 7 | 0.76 (0.36-1.61) | 0.77 (0.5) | 0.77 (0.36) | -0.38 (-1.37) |
| Hepatobiliary disorders | 5 | 1.21 (0.5-2.92) | 1.21 (0.18) | 1.21 (0.5) | 0.27 (-0.97) |
| Renal and urinary disorders | 5 | 0.69 (0.29-1.67) | 0.69 (0.69) | 0.69 (0.29) | -0.53 (-1.64) |
| Eye disorders | 4 | 0.41 (0.15-1.1) | 0.42 (3.34) | 0.42 (0.16) | -1.26 (-2.38) |
| Vascular disorders | 3 | 0.35 (0.11-1.1) | 0.36 (3.56) | 0.36 (0.11) | -1.49 (-2.69) |
| Ear and labyrinth disorders | 2 | 1.04 (0.26-4.16) | 1.04 (0) | 1.04 (0.26) | 0.05 (-1.64) |
| Immune system disorders | 2 | 0.36 (0.09-1.45) | 0.37 (2.23) | 0.37 (0.09) | -1.45 (-2.78) |
| Product issues | 0 | - | - | - | - |
| Reproductive system and breast disorders | 0 | - | - | - | - |
| Social circumstances | 0 | - | - | - | - |
| Congenital, familial and genetic disorders | 0 | - | - | - | - |
| Endocrine disorders | 0 | - | - | - | - |
| Pregnancy, puerperium and perinatal conditions | 0 | - | - | - | - |
| This table presents the number of individual case safety reports and quantitative signal detection metrics for four FDA-approved Bruton tyrosine kinase (BTK) inhibitors in the FDA Adverse Event Reporting System (FAERS) at the SOC level. Metrics include: Reporting Odds Ratio (ROR) with 95% confidence interval (95% CI); Proportional Reporting Ratio (PRR) with chi-square test statistic (χ²); Empirical Bayes Geometric Mean (EBGM) with 5th percentile (EBGM_05_); Information Component (IC) with 25th percentile (IC_025_); "-": the corresponding signal detection metric could not be calculated. | | | | | |

Supplementary Table 6. Comparative Summary of Core SOC/PT Signals for Four FDA-Approved BTK Inhibitors in FAERS

| **SOC** | **Common Core PTs** | **Drug-Specific High-Risk PTs** |
| --- | --- | --- |
| Blood and lymphatic system disorders | Abnormal white blood cell count, Decreased platelet count | Zanubrutinib: Myelosuppression  (ROR = 16.24) |
| Cardiac disorders | Atrial fibrillation | Ibrutinib: Atrial fibrillation  (ROR = 9.8) |
| Infections and Infestations | Pneumonia | Pirtobrutinib: Pneumonia  (ROR = 4.53) |
| General Disorders and Administration Site Conditions | Death | No unique high-risk PTs identified |
| Skin and subcutaneous tissue disorders | Contusion, Rash | Zanubrutinib: Haemorrhage subcutaneous (ROR = 146.81) |

Supplementary Table 7. High- and Moderate-Priority AEs Among Top 30 PTs for Four FDA-Approved BTK Inhibitors in FAERS

| **BTK Inhibitor** | **High Priority PT** | **Moderate Priority PTs** |
| --- | --- | --- |
| Ibrutinib | Death | Atrial fibrillation, Pneumonia, Contusion, Fall, Incorrect dose administered |
| Acalabrutinib | Death | Headache, Malignant neoplasm progression, Contusion, Fall, Product dose omission issue, Atrial fibrillation, Cerebrovascular accident |
| Zanubrutinib | None | Off label use, Contusion, Rash, Disease progression, Myelosuppression, White blood cell count decreased, Platelet count decreased, Atrial fibrillation |
| Pirtobrutinib | Malignant neoplasm progression | White blood cell count increased, Pneumonia, Anaemia, Pyrexia, Contusion, Hospitalisation, Illness, Cardiac failure, Febrile neutropenia, Sepsis |

Supplementary Table 8-A. Comparison of Shared PT Signals and Sensitivity Analysis for the Top 30 Adverse Events of Ibrutinib

| **Shared PTs of Ibrutinib** | **Full-Cycle ROR (95% CI)** | **2-Year Window ROR (95% CI)** | **ROR Difference Rate (%)** | **95% CI Overlap** | **Stability Determination** |
| --- | --- | --- | --- | --- | --- |
| death | 2.58 (2.52-2.64) | 3.90 (3.72-4.08) | +51.16% | No | Meaningful change |
| off label use | 1.34 (1.30-1.38) | 0.51 (0.47-0.56) | -61.94% | No | Meaningful change |
| fatigue | 1.52 (1.47-1.56) | 1.18 (1.09-1.28) | -22.37% | Yes | Basically stable |
| diarrhoea | 1.60 (1.55-1.65) | 1.38 (1.27-1.50) | -13.75% | Yes | Basically stable |
| atrial fibrillation | 9.80 (9.44-10.17) | 10.29 (9.42-11.24) | +5.00% | Yes | stable |
| pneumonia | 2.44 (2.35-2.53) | 2.19 (1.99-2.40) | -10.25% | Yes | Basically stable |
| contusion | 8.30 (7.98-8.63) | 6.63 (5.98-7.36) | -20.12% | No | Meaningful change |
| fall | 2.07 (1.99-2.16) | 2.62 (2.39-2.87) | +26.57% | No | Meaningful change |
| asthenia | 1.67 (1.60-1.75) | 1.49 (1.34-1.67) | -10.78% | Yes | Basically stable |
| arthralgia | 1.38 (1.32-1.44) | 1.20 (1.09-1.34) | -13.04% | Yes | Basically stable |
| nausea | 0.73 (0.70-0.76) | 0.64 (0.57-0.72) | -12.33% | Yes | Basically stable |
| rash | 1.28 (1.23-1.34) | 1.13 (1.01-1.26) | -11.72% | Yes | Basically stable |
| muscle spasms | 2.65 (2.52-2.78) | 2.86 (2.54-3.22) | +7.92% | Yes | stable |
| dizziness | 0.94 (0.89-0.99) | 0.90 (0.79-1.02) | -4.26% | Yes | stable |
| hospitalisation | 2.59 (2.46-2.72) | 2.71 (2.43-3.02) | +4.63% | Yes | stable |
| haemorrhage | 4.18 (3.97-4.41) | 4.01 (3.54-4.54) | -4.07% | Yes | stable |
| pain | 0.65 (0.62-0.68) | 0.98 (0.88-1.09) | +50.77% | No | Meaningful change |
| peripheral swelling | 2.20 (2.09-2.32) | 1.86 (1.62-2.14) | -15.45% | Yes | Basically stable |
| covid-19 | 1.75 (1.66-1.85) | 1.71 (1.56-1.87) | -2.29% | Yes | stable |
| dyspnoea | 0.71 (0.68-0.75) | 0.60 (0.52-0.69) | -15.49% | Yes | Basically stable |
| pyrexia | 1.20 (1.14-1.27) | 0.86 (0.74-1.00) | -28.33% | No | Unstable |
| white blood cell count increased | 12.06 (11.41-12.75) | 11.84 (10.33-13.57) | -1.82% | Yes | stable |
| headache | 0.61 (0.57-0.64) | 0.61 (0.54-0.70) | 0.00% | Yes | stable |
| hypertension | 1.78 (1.68-1.88) | 2.76 (2.48-3.06) | +55.06% | No | Meaningful change |
| urinary tract infection | 2.03 (1.92-2.15) | 2.13 (1.87-2.42) | +4.93% | Yes | stable |

Supplementary Table 8-B. Comparison of Shared PT Signals and Sensitivity Analysis for the Top 30 Adverse Events of Acalabrutinib

| **Shared PTs of Acalabrutinib** | **Full-Cycle ROR (95% CI)** | **2-Year Window ROR (95% CI)** | **ROR Difference Rate (%)** | **95% CI Overlap** | **Stability Determination** |
| --- | --- | --- | --- | --- | --- |
| death | 7.09 (6.75-7.45) | 5.68 (5.29-6.10) | -19.89% | Yes | Basically stable |
| headache | 2.66 (2.42-2.91) | 2.66 (2.36-3.00) | 0.00% | Yes | stable |
| fatigue | 1.69 (1.53-1.87) | 1.68 (1.48-1.90) | -0.59% | Yes | stable |
| product dose omission issue | 2.23 (1.98-2.51) | 2.00 (1.76-2.28) | -10.31% | Yes | Basically stable |
| malignant neoplasm progression | 7.34 (6.49-8.31) | 6.04 (5.04-7.22) | -17.71% | Yes | Basically stable |
| diarrhoea | 1.23 (1.09-1.40) | 1.20 (1.02-1.41) | -2.44% | Yes | stable |
| contusion | 8.38 (7.34-9.57) | 8.55 (7.25-10.09) | +2.03% | No | Meaningful change |
| dyspnoea | 1.28 (1.12-1.47) | 1.43 (1.21-1.70) | +11.72% | No | Meaningful change |
| fall | 2.18 (1.90-2.51) | 2.88 (2.46-3.37) | +32.11% | No | Meaningful change |
| asthenia | 1.66 (1.43-1.92) | 1.65 (1.36-2.00) | -0.60% | Yes | stable |
| pneumonia | 1.79 (1.55-2.08) | 1.70 (1.40-2.06) | -5.03% | Yes | stable |
| pain | 0.86 (0.74-0.99) | 1.07 (0.89-1.30) | +24.42% | No | Meaningful change |
| atrial fibrillation | 6.06 (5.21-7.05) | 6.02 (4.91-7.37) | -0.66% | Yes | stable |
| arthralgia | 1.27 (1.08-1.48) | 1.10 (0.90-1.35) | -13.39% | Yes | Basically stable |
| nausea | 0.70 (0.59-0.82) | 0.53 (0.42-0.67) | -24.29% | Yes | Basically stable |
| rash | 1.10 (0.93-1.29) | 0.85 (0.67-1.08) | -22.73% | Yes | Basically stable |
| dizziness | 1.08 (0.92-1.28) | 1.22 (1.00-1.49) | +12.96% | Yes | Basically stable |
| covid-19 | 1.51 (1.29-1.78) | 0.95 (0.76-1.19) | -37.09% | Yes | Unstable |
| pyrexia | 1.40 (1.18-1.65) | 1.17 (0.92-1.47) | -16.43% | Yes | Basically stable |
| myalgia | 2.92 (2.46-3.46) | 2.92 (2.34-3.64) | 0.00% | Yes | stable |
| haemorrhage | 4.15 (3.47-4.97) | 4.15 (3.47-4.97) | 0.00% | Yes | stable |
| white blood cell count increased | 12.93 (10.80-15.47) | 11.40 (8.90-14.59) | -11.83% | Yes | Basically stable |
| anaemia | 2.32 (1.93-2.78) | 2.24 (1.76-2.85) | -3.45% | Yes | stable |
| peripheral swelling | 1.96 (1.63-2.37) | 2.54 (2.05-3.16) | +29.59% | Yes | Basically stable |
| platelet count decreased | 3.00 (2.46-3.67) | 2.94 (2.28-3.79) | -2.00% | Yes | stable |
| lymphadenopathy | 9.88 (8.09-12.07) | 9.51 (7.34-12.33) | -3.74% | Yes | stable |
| cerebrovascular accident | 2.60 (2.12-3.18) | 3.97 (3.18-4.95) | +52.69% | No | Meaningful change |

Supplementary Table 8-C. Comparison of Shared PT Signals and Sensitivity Analysis for the Top 30 Adverse Events of Zanubrutinib

| **Shared PTs of Zanubrutinib** | **Full-Cycle ROR**  **(95% CI)** | **2-Year Window ROR**  **(95% CI)** | **ROR Difference Rate (%)** | **95% CI Overlap** | **Stability Determination** |
| --- | --- | --- | --- | --- | --- |
| off label use | 2.26 (1.99-2.57) | 1.40 (1.18-1.65) | -38.05% | No | Meaningful change |
| contusion | 14.81 (12.33-17.79) | 13.02 (10.46-16.21) | -12.09% | Yes | Basically stable |
| rash | 2.46 (2.02-3.01) | 2.34 (1.86-2.96) | -4.88% | Yes | stable |
| fatigue | 1.29 (1.05-1.58) | 1.21 (0.95-1.53) | -6.20% | Yes | stable |
| disease progression | 7.79 (6.33-9.58) | 7.70 (6.13-9.66) | -1.15% | Yes | stable |
| myelosuppression | 16.24 (13.12-20.11) | 11.37 (8.91-14.51) | -30.00% | Yes | Basically stable |
| death | 0.95 (0.76-1.19) | 0.85 (0.64-1.12) | -10.53% | Yes | Basically stable |
| white blood cell count decreased | 6.17 (4.84-7.86) | 7.20 (5.54-9.35) | +16.78% | Yes | Basically stable |
| product dose omission issue | 1.20 (0.93-1.55) | 1.02 (0.77-1.36) | -15.00% | Yes | Basically stable |
| diarrhoea | 0.97 (0.75-1.26) | 0.84 (0.61-1.15) | -13.40% | Yes | Basically stable |
| platelet count decreased | 5.73 (4.41-7.44) | 5.21 (3.81-7.11) | -9.08% | Yes | stable |
| asthenia | 1.69 (1.28-2.21) | 1.40 (1.00-1.96) | -17.16% | Yes | Basically stable |
| dizziness | 1.30 (0.99-1.72) | 1.29 (0.94-1.78) | -0.77% | Yes | stable |
| nausea | 0.78 (0.59-1.03) | 0.69 (0.50-0.97) | -11.54% | Yes | Basically stable |
| pneumonia | 1.69 (1.28-2.25) | 1.62 (1.18-2.23) | -4.14% | Yes | stable |
| prescribed underdose | 21.10 (15.87-28.05) | 17.98 (12.46-25.94) | -14.80% | Yes | Basically stable |
| atrial fibrillation | 5.23 (3.89-7.04) | 5.40 (3.81-7.64) | +3.25% | Yes | stable |
| underdose | 7.33 (5.45-9.87) | 7.80 (5.54-11.00) | +6.41% | Yes | stable |
| dyspnoea | 0.90 (0.67-1.21) | 0.82 (0.57-1.17) | -8.89% | Yes | stable |
| covid-19 | 1.11 (0.82-1.50) | 1.01 (0.71-1.43) | -9.01% | Yes | stable |
| cough | 1.54 (1.14-2.09) | 1.34 (0.93-1.92) | -12.99% | Yes | Basically stable |
| pyrexia | 1.36 (0.99-1.85) | 1.26 (0.88-1.82) | -7.35% | Yes | stable |
| white blood cell count increased | 14.52 (10.64-19.83) | 13.12 (9.04-19.05) | -9.64% | Yes | stable |
| arthralgia | 1.00 (0.73-1.36) | 0.95 (0.67-1.35) | -5.00% | Yes | stable |
| haemorrhage subcutaneous | 146.81 (106.14-203.07) | 170.73 (117.70-247.64) | +16.30% | Yes | Basically stable |
| fall | 1.33 (0.97-1.84) | 1.33 (0.97-1.84) | 0.00% | Yes | stable |
| urinary tract infection | 2.28 (1.64-3.16) | 2.18 (1.50-3.16) | -4.39% | Yes | stable |
| drug ineffective | 0.28 (0.20-0.38) | 0.32 (0.22-0.46) | +14.29% | Yes | Basically stable |

For shared preferred terms (PTs) among the top 30 PTs by occurrence count, signal stability was assessed using the ROR difference rate and 95% confidence interval (CI) overlap. The ROR difference rate was calculated as: ROR difference rate = [(ROR of 2-year window − ROR of full cycle) / ROR of full cycle] × 100%. Signal stability was further determined by assessing the overlap of 95% confidence intervals (95% CIs). Based on predefined stability criteria: Stable: Absolute ROR difference rate <10% with overlapping 95% CIs; Basically stable: ROR difference rate of 10%-30% or overlapping 95% CIs; Meaningful change: ROR difference rate >30%, Unstable: Non-overlapping 95% CIs with a change in signal direction.

Supplementary Table 9-A. Signal Detection Metrics for the Top 30 Most Frequently Reported Preferred Terms of Ibrutinib Stratified by Subgroup

| **No** | **Ibrutinib** | **Overall ROR (95% CI)** | **Serious AE ROR (95% CI)** | **First-line ROR (95% CI)** |
| --- | --- | --- | --- | --- |
| 1 | death | 2.58 (2.52-2.64) | 3.27 (3.19-3.34) | 3.04 (2.97-3.11) |
| 2 | off label use | 1.34 (1.3-1.38) | 1.14 (1.1-1.18) | 1.18 (1.14-1.22) |
| 3 | fatigue | 1.52 (1.47-1.56) | 1.18 (1.14-1.23) | 1.45 (1.41-1.51) |
| 4 | diarrhoea | 1.6 (1.55-1.65) | 1.33 (1.27-1.38) | 1.53 (1.48-1.59) |
| 5 | atrial fibrillation | 9.8 (9.44-10.17) | 10.47 (10.05-10.89) | 9.83 (9.44-10.24) |
| 6 | incorrect dose administered | 3.53 (3.4-3.67) | 2.58 (2.46-2.71) | 3.2 (3.06-3.34) |
| 7 | pneumonia | 2.44 (2.35-2.53) | 2.88 (2.76-2.99) | 2.43 (2.32-2.53) |
| 8 | contusion | 8.3 (7.98-8.63) | 6.66 (6.34-6.99) | 7.71 (7.38-8.06) |
| 9 | fall | 2.07 (1.99-2.16) | 2.37 (2.27-2.48) | 2.15 (2.06-2.25) |
| 10 | asthenia | 1.67 (1.6-1.75) | 1.67 (1.59-1.75) | 1.61 (1.53-1.69) |
| 11 | arthralgia | 1.38 (1.32-1.44) | 1.1 (1.04-1.16) | 1.3 (1.23-1.36) |
| 12 | nausea | 0.73 (0.7-0.76) | 0.61 (0.58-0.65) | 0.69 (0.65-0.72) |
| 13 | rash | 1.28 (1.23-1.34) | 0.85 (0.8-0.9) | 1.26 (1.2-1.33) |
| 14 | muscle spasms | 2.65 (2.52-2.78) | 1.86 (1.74-1.98) | 2.44 (2.31-2.58) |
| 15 | dizziness | 0.94 (0.89-0.99) | 0.8 (0.75-0.85) | 0.88 (0.83-0.93) |
| 16 | hospitalisation | 2.59 (2.46-2.72) | 3.25 (3.08-3.42) | 2.89 (2.75-3.05) |
| 17 | haemorrhage | 4.18 (3.97-4.41) | 4.49 (4.25-4.75) | 4.19 (3.96-4.44) |
| 18 | pain | 0.65 (0.62-0.68) | 0.55 (0.52-0.59) | 0.67 (0.63-0.7) |
| 19 | peripheral swelling | 2.2 (2.09-2.32) | 1.91 (1.8-2.04) | 2.08 (1.97-2.21) |
| 20 | platelet count decreased | 3.89 (3.69-4.11) | 3.81 (3.58-4.04) | 3.51 (3.3-3.73) |
| 21 | covid-19 | 1.75 (1.66-1.85) | 1.42 (1.33-1.52) | 2.1 (1.99-2.21) |
| 22 | dyspnoea | 0.71 (0.68-0.75) | 0.76 (0.72-0.81) | 0.72 (0.68-0.76) |
| 23 | pyrexia | 1.2 (1.14-1.27) | 1.27 (1.19-1.34) | 1.16 (1.09-1.23) |
| 24 | white blood cell count increased | 12.06 (11.41-12.75) | 10.96 (10.28-11.69) | 11.41 (10.72-12.13) |
| 25 | headache | 0.61 (0.57-0.64) | 0.52(0.49-0.56) | 0.59 (0.56-0.63) |
| 26 | hypertension | 1.78 (1.68-1.88) | 1.68(1.58-1.80) | 1.88 (1.77-2) |
| 27 | urinary tract infection | 2.03 (1.92-2.15) | 2.04 (1.91-2.17) | 2.09 (1.96-2.22) |
| 28 | disease progression | 2.93 (2.77-3.11) | 3.17 (2.97-3.37) | 2.81 (2.64-3) |
| 29 | surgery | 6.19 (5.84-6.57) | 7.74 (7.3-8.21) | 7.08 (6.67-7.52) |
| 30 | weight decreased | 1.2 (1.13-1.27) | 3.27 (3.19-3.34) | 3.04 (2.97-3.11) |

Supplementary Table 9-B. Signal Detection Metrics for the Top 30 Most Frequently Reported Preferred Terms of Acalabrutinib Stratified by Subgroup

| **No** | **Acalabrutinib** | **Overall ROR (95% CI)** | **Serious AE ROR (95% CI)** | **First-line ROR (95% CI)** |
| --- | --- | --- | --- | --- |
| 1 | death | 7.09 (6.75-7.45) | 8.86 (8.43-9.32) | 7.11 (6.77-7.46) |
| 2 | headache | 2.66 (2.42-2.91) | 1.63 (1.43-1.85) | 2.66 (2.43-2.92) |
| 3 | fatigue | 1.69 (1.53-1.87) | 1.27 (1.12-1.44) | 1.69 (1.54-1.87) |
| 4 | product dose omission issue | 2.23 (1.98-2.51) | 2.40 (2.12-2.72) | 2.23 (1.98-2.51) |
| 5 | malignant neoplasm progression | 7.34 (6.49-8.31) | 9.00 (7.95-10.18) | 7.36 (6.50-8.33) |
| 6 | diarrhoea | 1.23 (1.09-1.40) | 0.96 (0.82-1.12) | 1.23 (1.08-1.39) |
| 7 | contusion | 8.38 (7.34-9.57) | 6.07 (5.12-7.21) | 8.36 (7.32-9.55) |
| 8 | dyspnoea | 1.28 (1.12-1.47) | 1.40 (1.21-1.62) | 1.29 (1.12-1.47) |
| 9 | fall | 2.18 (1.90-2.51) | 2.64 (2.29-3.03) | 2.19 (1.91-2.51) |
| 10 | asthenia | 1.66 (1.43-1.92) | 1.71 (1.45-2.01) | 1.65 (1.42-1.92) |
| 11 | pneumonia | 1.79 (1.55-2.08) | 2.08 (1.79-2.43) | 1.79 (1.54-2.08) |
| 12 | pain | 0.86 (0.74-0.99) | 0.70 (0.58-0.84) | 0.86 (0.74-1.00) |
| 13 | atrial fibrillation | 6.06 (5.21-7.05) | 6.71 (5.72-7.87) | 6.01 (5.16-7.00) |
| 14 | arthralgia | 1.27 (1.08-1.48) | 0.83 (0.67-1.03) | 1.27 (1.09-1.48) |
| 15 | nausea | 0.70 (0.59-0.82) | 0.58 (0.48-0.70) | 0.69 (0.59-0.81) |
| 16 | rash | 1.10 (0.93-1.29) | 0.81 (0.66-1.00) | 1.10 (0.94-1.29) |
| 17 | dizziness | 1.08 (0.92-1.28) | 0.96 (0.79-1.16) | 1.08 (0.92-1.27) |
| 18 | covid-19 | 1.51 (1.29-1.78) | 1.65 (1.39-1.96) | 1.52 (1.29-1.79) |
| 19 | pyrexia | 1.40 (1.18-1.65) | 1.47 (1.22-1.76) | 1.40 (1.18-1.66) |
| 20 | myalgia | 2.92 (2.46-3.46) | 1.75 (1.37-2.23) | 2.92 (2.46-3.47) |
| 21 | haemorrhage | 4.15 (3.47-4.97) | 4.49 (3.71-5.43) | 4.16 (3.48-4.98) |
| 22 | white blood cell count increased | 12.93 (10.80-15.47) | 11.19 (9.05-13.84) | 12.96 (10.83-15.51) |
| 23 | anaemia | 2.32 (1.93-2.78) | 2.59 (2.15-3.13) | 2.30 (1.92-2.76) |
| 24 | peripheral swelling | 1.96 (1.63-2.37) | 1.73 (1.39-2.15) | 1.97 (1.63-2.37) |
| 25 | drug ineffective | 0.25 (0.21-0.30) | 0.27 (0.22-0.33) | 0.25 (0.21-0.30) |
| 26 | off label use | 0.30 (0.25-0.37) | 0.29 (0.24-0.36) | 0.30 (0.25-0.36) |
| 27 | platelet count decreased | 3.00 (2.46-3.67) | 3.03 (2.43-3.77) | 3.01 (2.47-3.68) |
| 28 | lymphadenopathy | 9.88 (8.09-12.07) | 9.95 (7.98-12.40) | 9.91 (8.11-12.10) |
| 29 | cerebrovascular accident | 2.60 (2.12-3.18) | 3.18 (2.60-3.89) | 2.61 (2.13-3.19) |
| 30 | haemoglobin decreased | 3.31 (2.69-4.07) | 3.46 (2.77-4.33) | 3.32 (2.70-4.08) |

Supplementary Table 9-C. Signal Detection Metrics for the Top 30 Most Frequently Reported Preferred Terms of Zanubrutinib Stratified by Subgroup

| **No** | **Zanubrutinib** | **Overall ROR (95% CI)** | **Serious AE ROR (95% CI)** | **First-line ROR (95% CI)** |
| --- | --- | --- | --- | --- |
| 1 | off label use | 2.26 (1.99-2.57) | 2.34 (2.02-2.71) | 2.26 (1.99-2.57) |
| 2 | contusion | 14.81 (12.33-17.79) | 9.43 (7.26-12.25) | 14.85 (12.36-17.84) |
| 3 | rash | 2.46 (2.02-3.01) | 1.48 (1.10-1.98) | 2.47 (2.02-3.01) |
| 4 | fatigue | 1.29 (1.05-1.58) | 0.98 (0.75-1.28) | 1.29 (1.05-1.59) |
| 5 | disease progression | 7.79 (6.33-9.58) | 10.32 (8.38-12.71) | 7.81 (6.34-9.60) |
| 6 | myelosuppression | 16.24 (13.12-20.11) | 21.52 (17.38-26.66) | 16.09 (12.98-19.94) |
| 7 | death | 0.95 (0.76-1.19) | 1.25 (1.00-1.57) | 0.95 (0.76-1.19) |
| 8 | white blood cell count decreased | 6.17 (4.84-7.86) | 7.28 (5.63-9.42) | 6.18 (4.85-7.88) |
| 9 | product dose omission issue | 1.2 (0.93-1.55) | 1.06 (0.78-1.45) | 1.20 (0.93-1.55) |
| 10 | diarrhoea | 0.97 (0.75-1.26) | 0.84 (0.61-1.15) | 0.97 (0.75-1.26) |
| 11 | platelet count decreased | 5.73 (4.41-7.44) | 5.83 (4.33-7.85) | 5.74 (4.42-7.46) |
| 12 | asthenia | 1.69 (1.28-2.21) | 1.93 (1.44-2.58) | 1.69 (1.29-2.22) |
| 13 | dizziness | 1.3 (0.99-1.72) | 1.01 (0.70-1.44) | 1.31 (0.99-1.72) |
| 14 | nausea | 0.78 (0.59-1.03) | 0.72 (0.51-1.00) | 0.78 (0.59-1.03) |
| 15 | pneumonia | 1.69 (1.28-2.25) | 2.24 (1.69-2.97) | 1.70 (1.28-2.25) |
| 16 | prescribed underdose | 21.1 (15.87-28.05) | 19.08 (13.54-26.89) | 21.14 (15.90-28.11) |
| 17 | atrial fibrillation | 5.23 (3.89-7.04) | 6.76 (5.01-9.13) | 5.25 (3.90-7.06) |
| 18 | underdose | 7.33 (5.45-9.87) | 6.36 (4.42-9.17) | 7.35 (5.46-9.89) |
| 19 | dyspnoea | 0.9 (0.67-1.21) | 0.99 (0.72-1.38) | 0.90 (0.67-1.22) |
| 20 | covid-19 | 1.11 (0.82-1.5) | 1.09 (0.77-1.55) | 1.12 (0.83-1.51) |
| 21 | cough | 1.54 (1.14-2.09) | 1.40 (0.97-2.02) | 1.54 (1.14-2.09) |
| 22 | pyrexia | 1.36 (0.99-1.85) | 1.34 (0.94-1.92) | 1.36 (1.00-1.85) |
| 23 | white blood cell count increased | 14.52 (10.64-19.83) | 9.54 (6.14-14.80) | 14.56 (10.66-19.88) |
| 24 | arthralgia | 1 (0.73-1.36) | 0.72 (0.47-1.10) | 1.00 (0.73-1.36) |
| 25 | pruritus | 1.05 (0.76-1.45) | 1.06 (0.73-1.52) | 1.05 (0.77-1.45) |
| 26 | haemorrhage subcutaneous | 146.81 (106.14-203.07) | 178.07 (127.03-249.64) | 147.15 (106.38-203.54) |
| 27 | fall | 1.33 (0.97-1.84) | 1.52 (1.08-2.16) | 1.34 (0.97-1.85) |
| 28 | urinary tract infection | 2.28 (1.64-3.16) | 2.67 (1.89-3.78) | 2.15 (1.54-3.02) |
| 29 | drug ineffective | 0.28 (0.2-0.38) | 0.28 (0.19-0.41) | 0.28 (0.20-0.38) |
| 30 | headache | 0.70 (0.5-0.97) | 0.38(0.23-0.63) | 0.70 (0.50-0.97) |

ROR: Reporting Odds Ratio

Supplementary Table 10. Disproportionality Analysis for Selected Negative Control Outcomes

| **PT** | **Ibrutinib ROR (95% CI)** | **Acalabrutinib ROR (95% CI)** | **Zanubrutinib ROR (95% CI)** | **Pirtobrutinib ROR (95% CI)** |
| --- | --- | --- | --- | --- |
| osteoarthritis | 0.74 (0.61-0.90) | 1.91 (0.72-5.09) | 1.56 (0.22-11.05) | 3.09 (0.43-22.01) |
| acne | 0.54 (0.46-0.62) | 0.21 (0.10-0.43) | 0.12 (0.02-0.88) | No reports |
| hypoacusis | 0.95 (0.81-1.11) | 1.45 (0.97-2.17) | 0.97 (0.40-2.33) | No reports |
| tinnitus | 0.66 (0.54-0.81) | 0.89 (0.49-1.60) | 0.83 (0.27-2.56) | No reports |
| diverticulitis | 1.08 (0.89-1.32) | 0.59 (0.24-1.41) | 0.82 (0.21-3.29) | No reports |

ROR: Reporting Odds Ratio. A statistically significant positive disproportionality signal is defined as an ROR with a 95% CI lower bound > 1. In this table, none of the estimates for the negative control outcomes meet this criterion.

Supplementary Table 11-A. Association Analysis Between Preferred Terms and Fatal Outcomes for Ibrutinib

| **Ibrutinib PT** | **a** | **b** | **c** | **d** | **p value** | **chisq stat** | **adj p value** | **bonferroni p value** |
| --- | --- | --- | --- | --- | --- | --- | --- | --- |
| off label use | 472 | 17395 | 4074 | 187705 | 0.00 | 20.38 | 0.00 | 0.01 |
| disease progression | 354 | 17513 | 822 | 190957 | 0.00 | 703.63 | 0.00 | 0.00 |
| pneumonia | 309 | 17558 | 2342 | 189437 | 0.00 | 33.41 | 0.00 | 0.00 |
| chronic lymphocytic leukaemia | 184 | 17683 | 395 | 191384 | 0.00 | 399.81 | 0.00 | 0.00 |
| atrial fibrillation | 182 | 17685 | 2759 | 189020 | 0.00 | 20.54 | 0.00 | 0.01 |
| incorrect dose administered | 171 | 17696 | 2542 | 189237 | 0.00 | 17.08 | 0.00 | 0.05 |
| sepsis | 163 | 17704 | 552 | 191227 | 0.00 | 185.69 | 0.00 | 0.00 |
| covid-19 | 161 | 17706 | 1199 | 190580 | 0.00 | 18.88 | 0.00 | 0.02 |
| diarrhoea | 148 | 17719 | 3426 | 188353 | 0.00 | 88.96 | 0.00 | 0.00 |
| fall | 129 | 17738 | 2134 | 189645 | 0.00 | 23.01 | 0.00 | 0.00 |
| product use in unapproved indication | 116 | 17751 | 612 | 191167 | 0.00 | 50.52 | 0.00 | 0.00 |
| respiratory failure | 115 | 17752 | 215 | 191564 | 0.00 | 290.45 | 0.00 | 0.00 |
| thrombocytopenia | 109 | 17758 | 618 | 191161 | 0.00 | 38.35 | 0.00 | 0.00 |
| infection | 102 | 17765 | 776 | 191003 | 0.00 | 10.44 | 0.01 | 1.00 |
| myocardial infarction | 100 | 17767 | 489 | 191290 | 0.00 | 53.08 | 0.00 | 0.00 |
| pyrexia | 100 | 17767 | 1232 | 190547 | 0.20 | 1.64 | 0.45 | 1.00 |
| asthenia | 97 | 17770 | 1963 | 189816 | 0.00 | 38.32 | 0.00 | 0.00 |
| anaemia | 93 | 17774 | 946 | 190833 | 0.66 | 0.19 | 0.82 | 1.00 |
| haemorrhage | 93 | 17774 | 1344 | 190435 | 0.01 | 7.54 | 0.03 | 1.00 |
| cardiac failure | 91 | 17776 | 391 | 191388 | 0.00 | 65.15 | 0.00 | 0.00 |

Supplementary Table 11-B. Association Analysis Between Preferred Terms and Fatal Outcomes for Acalabrutinib

| **Acalabrutinib PT** | **a** | **b** | **c** | **d** | **p value** | **chisq stat** | **adj p value** | **bonferroni p value** |
| --- | --- | --- | --- | --- | --- | --- | --- | --- |
| covid-19 | 34 | 2477 | 112 | 16188 | 0.00 | 11.72 | 0.01 | 0.20 |
| pneumonia | 33 | 2478 | 141 | 16159 | 0.04 | 4.31 | 0.23 | 1.00 |
| malignant neoplasm progression | 30 | 2481 | 225 | 16075 | 0.51 | 0.43 | 0.74 | 1.00 |
| off label use | 16 | 2495 | 92 | 16208 | 0.76 | 0.09 | 0.92 | 1.00 |
| fatigue | 15 | 2496 | 389 | 15911 | 0.00 | 32.30 | 0.00 | 0.00 |
| sepsis | 11 | 2500 | 33 | 16267 | 0.04 | 4.22 | 0.24 | 1.00 |
| asthenia | 9 | 2502 | 165 | 16135 | 0.00 | 9.45 | 0.03 | 0.67 |
| fall | 9 | 2502 | 197 | 16103 | 0.00 | 13.75 | 0.00 | 0.07 |
| general physical health deterioration | 9 | 2502 | 18 | 16282 | 0.01 | - | 0.07 | 1.00 |
| diarrhoea | 8 | 2503 | 238 | 16062 | 0.00 | 21.09 | 0.00 | 0.00 |
| headache | 8 | 2503 | 457 | 15843 | 0.00 | 54.71 | 0.00 | 0.00 |
| cardiac failure | 7 | 2504 | 42 | 16258 | 1.00 | 0.00 | 1.00 | 1.00 |
| dyspnoea | 7 | 2504 | 200 | 16100 | 0.00 | 17.12 | 0.00 | 0.01 |
| infection | 7 | 2504 | 75 | 16225 | 0.26 | 1.26 | 0.58 | 1.00 |
| nausea | 7 | 2504 | 146 | 16154 | 0.00 | 9.51 | 0.03 | 0.65 |
| septic shock | 7 | 2504 | 11 | 16289 | 0.01 | - | 0.07 | 1.00 |
| cardiac arrest | 6 | 2505 | 10 | 16290 | 0.01 | - | 0.12 | 1.00 |
| cerebrovascular accident | 6 | 2505 | 89 | 16211 | 0.06 | 3.49 | 0.29 | 1.00 |
| decreased appetite | 6 | 2505 | 73 | 16227 | 0.18 | 1.80 | 0.54 | 1.00 |
| disease progression | 6 | 2505 | 59 | 16241 | 0.43 | 0.63 | 0.69 | 1.00 |
| a: number of cases with the PT in the death group; b: number of cases without the PT in the death group; c: number of cases with the PT in the non-death group; d: number of cases without the PT in the non-death group; p-value: statistical significance (p-value < 0.05 indicates a significant difference in PT occurrence frequency between the death group and non-death group); chisq stat: chi-square value (reflects the strength of frequency difference between groups; larger values indicate more significant differences); adj p value/bonferroni p value: p-values adjusted for multiple testing; - -: missing data. | | | | | | | | |

Supplementary Figure 1. Annual Trend in Adverse Event Reports for Four FDA-Approved BTK Inhibitors.

Supplementary Figure 2. Convergence plot of multiple imputation for zanubrutinib age groups. This figure illustrates the trends of the mean and standard deviation across age groups during the multiple imputation process for zanubrutinib data. The upper subplot displays the mean values, while the lower subplot shows the standard deviation, both plotted against the iteration number. As the iterations increased, the fluctuations in the mean and standard deviation for each age group converge and stabilize, demonstrating the robustness of the multiple imputation method.

Supplementary Figure 3. Gender distribution in zanubrutinib-treated patients after 10 rounds of multiple imputation. This figure illustrates the proportion of male (red) and female (cyan-blue) patients across 10 rounds of multiple imputation applied to zanubrutinib clinical data. Each bar represents the gender distribution for a single imputation round.

Supplementary Figure 4. Sex Distribution of FDA-Approved BTK Inhibitors in FAERS After Multiple Imputation

Supplementary Figure 5. Age Distribution of FDA-Approved BTK Inhibitors in FAERS After Multiple Imputation

Supplementary Figure 6. Line Chart Depicting the Percentage Trends in Overall AE Onset Time for the Four BTK Inhibitors.
